# Spatial determinants of tumor cell dedifferentiation and plasticity in primary cutaneous melanoma

**DOI:** 10.1101/2025.06.21.660851

**Published:** 2025-06-24

**Authors:** Tuulia Vallius, Yingxiao Shi, Edward Novikov, Shishir M Pant, Roxanne Pelletier, Yu-An Chen, Juliann B. Tefft, Ajit Nirmal Johnson, Zoltan Maliga, Guihong Wan, George Murphy, Sandro Santagata, Yevgeniy R Semenov, David Liu, Christine G Lian, Peter K Sorger

**Affiliations:** 1 Laboratory of Systems Pharmacology, Harvard Program in Therapeutic Science, Harvard Medical School, 200 Longwood Avenue, Boston, MA 02115; 2 Ludwig Center at Harvard; 3 Department of Systems Biology, Harvard Medical School, 200 Longwood Avenue, Boston, MA 02115.; 4 Department of Clinical Computational Oncology, Dana-Farber Cancer Institute, 450 Brookline Avenue, Boston, MA 02215; 5 Department of Dermatology, Brigham and Women’s Hospital, Harvard Medical School, 75 Francis Street, Boston, MA 02115; 6 Department of Dermatology, Massachusetts General Hospital; 7 Department of Medical Oncology, Dana-Farber Cancer Institute, 450 Brookline Avenue, Boston, MA 02215; 8 Program in Dermatopathology, Department of Pathology, Brigham and Women’s Hospital, Harvard Medical School, 75 Francis Street, Boston, MA 02115

**Keywords:** Cutaneous melanoma, multiplex imaging, spatial transcriptomics, cancer progression, dedifferentiation, lineage plasticity, cell state switch, spatial variance, intratumor heterogeneity, melanoma heterogeneity

## Abstract

Early detection of melanoma through skin surveillance is critical for preventing metastatic progression. Primary cutaneous melanomas at early stage offer a unique opportunity to uncover fundamental mechanisms of tumor initiation, progression, and immune surveillance, but detailed spatial profiling of early disease remains limited. Here we integrate high-plex cyclic immunofluorescence (CyCIF) imaging, spatial transcriptomics, and conventional histology to identify factors associated with de-differentiation and dermal invasion in early-stage melanomas. We demonstrate a high level of variability from one primary cancer to the next, from one 100–300 cell microregion to the next within a single cancer, and from one cell to the next within a microregion. Intra-tumoral heterogeneity is influenced by local features of the microenvironment including proximity to T and myeloid cells and to perivascular environments. Thus, tumor plasticity and spatial heterogeneity arise early in melanoma development, potentially allowing for competition among multiple tumor states during the emergence of invasive disease.

## STATEMENT OF SIGNIFICANCE

This study shows that tumor cell dedifferentiation and plasticity are locally patterned in primary human melanomas under the influence of microenvironment components such as myeloid cells and vasculature. As a result, neighborhoods in a single tumor can be as different from each other as in different tumors.

## INTRODUCTION

Cutaneous melanoma is the most aggressive type of skin cancer and its incidence is projected to nearly double between 2020 and 2040^1^. Melanoma remains a lethal disease in many patients, despite the approval of targeted therapies and immune checkpoint inhibitors (ICI)^2^. Early identification of disease involves routine skin surveillance^3^ followed by biopsy or excision of suspicious lesions for histopathological evaluation. This histopathological assessment is based on examination of hematoxylin and eosin (H&E) stained tissue sections prepared from formaldehyde-fixed paraffin embedded (FFPE) specimens, complemented in some cases by immunohistochemistry (IHC). Extensive analysis of this type of “traditional” histological data has led to highly refined morphological criteria for melanoma diagnosis and staging^4^.

In many cases, early melanomas can be cured by local excision. However, a substantial portion of metastatic disease occurs in patients who have undergone surgery with curative intent. Unfortunately, it remains difficult to predict which melanomas will recur, highlighting a substantial need for better diagnostic and risk stratification methods,^5^ ideally based on a better molecular understanding of the disease. Molecular profiling of melanoma using contemporary spatial transcriptomic and proteomic methods^6^ has mostly been performed in metastatic stage disease^7–9^, with primary melanomas remaining much less thoroughly studied^10^. In part, this is because formaldehyde fixation and small specimen size (along with a requirement to preserve tissue for patients who are still alive) make traditional RNA and scRNA sequencing difficult. However, the ready availability of FFPE specimens representing the earliest stages of disease offers a unique opportunity to study how cancers first arise and overcome immune surveillance and to potentially improve melanoma staging and risk assessment in the near term.

The initiation and progression of cutaneous melanoma is a multistep molecular process^11–15^ involving distinct histological stages that include (i) formation of precursor fields, which contain melanocytic cells with atypical morphologies and may or may not be associated with nevi, (ii) *melanoma in situ* (MIS), a pre-invasive state, (iii) locally invasive cancer exhibiting radial and/or vertical growth phases, (iv) locoregional metastasis (e.g. to draining lymph nodes), and (v) distant metastasis to the skin, brain, lungs, and liver. Cutaneous melanoma most commonly occurs in sun-exposed skin, which has a high mutational burden,^16^ and these melanomas have a characteristic “UV signature.”^17^ However, melanoma also occurs in non-sun-exposed skin.^18^ In some specimens, multiple stages of melanoma from precursor field to deeply invasive tumors can be found in a single tissue block, presenting an opportunity to study progression in a single individual.^10^

DNA sequencing has established an essential role for driver mutations in oncogenes such as *BRAF*, *NRAS* and *KIT* in melanoma initiation and metastasis^7,8,19,20^, and single cell sequencing and IHC have demonstrated a role for lineage plasticity and tumor cell dedifferentiation in therapy resistance, particularly in the metastatic setting^21–28^. Inflammatory microenvironments have been shown to induce cell plasticity^29–32^ resulting in a switch from a master transcriptional regulator *MITF*-driven proliferative melanocytic state to an invasive de-differentiated state characterized by expression of *NGFR*, *AXL*, and *SOX9*^7,22–24,33^. The relevance of these genetic and epigenetic changes to primary disease is only partly understood.

*MITF* plays a key role in the development of normal melanocytes by regulating expression of genes involved in melanosome formation and melanin biosynthesis. During tumor dedifferentiation and induction of an “epithelial-to-mesenchymal” transition (EMT), *MITF* expression decreases,^34^ leading to downregulation of transcriptional programs involved in melanosome maturation and pigment production. IHC of *MITF*-regulated proteins such as MART1^35^ and PMEL^36^ is used diagnostically to identify tumors of melanocytic lineage,^37–39^ as are lineage markers such as S100B (one of a family of related calcium binding proteins) and the SOX10 transcription factor^40^. However, staining patterns for these melanocytic markers are often heterogeneous^41^ both within a single specimen and across specimens of similar stages^39^. The current staging system (the AJCC 8th edition for primary cutaneous melanoma staging)^42^ therefore relies primarily on visual assessment of H&E-stained sections to score the depth of invasion (the Breslow depth), presence or absence of ulceration (which also has a macroscopic presentation), and whether lymph node metastases are present.^4,42^ The presence of tumor-adjacent lymphocytes (a brisk TIL response) is often scored as well, but the prognostic significance remains uncertain^43^. Expression of dedifferentiation or EMT-like transcriptomic programs is associated with poor survival in primary melanoma^34^, but no molecular or genetic features such as EMT are routinely evaluated in the primary disease setting. Thus, a substantial opportunity exists to profile the early-stage, primary melanoma samples to better predict biological behavior in clinical practice.

Metastatic melanoma is characterized by high intra-tumoral heterogeneity^9,44^ and this has also been observed in patient-derived xenografts^25^. Some studies (including those from our own laboratories) on limited numbers of primary melanomas have shown that dedifferentiation can be observed in pre-treatment and pre-metastatic stages^10,34,45–47^. However, these studies are limited by small sample sizes and incomplete sampling of progression stages. Thus, it is currently unknown when dedifferentiation and intratumoral heterogeneity first appear in primary melanomas or whether they arise later and how observed clinical features relate to heterogeneity at a molecular level.

In this paper we use spatial profiling and classical histopathology to study a cohort of 62 primary melanoma samples obtained from two dermatopathology services. Most of these specimens were selected to include multiple disease stages in a single tissue block. Using cyclic multiplexed immunofluorescence (CyCIF)^48^ imaging and GeoMX microregional transcriptomics,^49^ we characterize 313 distinct histological domains in these tumors ranging from adjacent normal skin to the cutaneous component of Stage III (metastatic) disease. We find that an overall trend from a differentiated to a de-differentiated state is overlaid by a high level of heterogeneity that plays out at three spatial scales: from one specimen to the next, from one 100–300 cell microregion to the next within a single specimen (as observed in 2D sections), and from one cell to the next within a microregion. Proximity to immune cells is one, but not the only, contributor to de-differentiation and local heterogeneity as quantified by spatial entropy. Fully dedifferentiated states, which resemble the embryonic progenitors of melanocytes (neural crest cells), are observed only at later stages of invasion, suggesting that they are consequences, not causes of tumor progression. These data show that the cell plasticity and dedifferentiation found in metastatic melanomas are also prominent features of early-stage disease.

## RESULTS

### Patient cohort and spatial data generation

**Figure 1.**
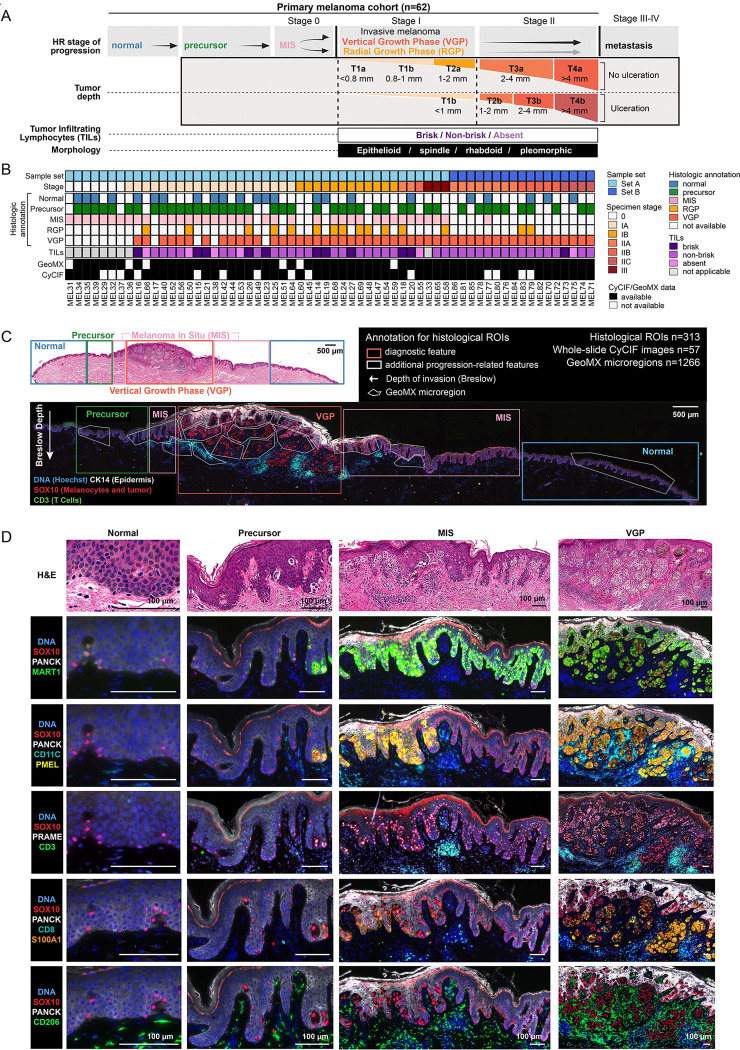
Multimodal profiling of primary melanoma. **A.** Summary of the current AJCC 8th edition for cutaneous melanoma staging^42^ based on features identified from H&Es. **B.** Overview of the primary melanoma cohort used for multimodal spatial profiling and their associated clinical (AJCC) and histological characteristics. (TILs: Tumor-infiltrating lymphocytes; normal skin; precursor; MIS: melanoma in situ; RGP: radial growth phase melanoma; VGP: vertical growth phase melanoma). **C.** Example specimen (MEL14) annotated with clinical histological stages (colored rectangles), as H&E (top) and CyCIF image of a serial section (bottom). CyCIF image shows a subset of markers: DNA (blue), CK14 (epidermis; white), SOX10 (melanocytes and tumor cells; red), and CD3 (T cells; green). White polygons overlaid represent the microregions (MRs) used for spatial transcriptomics (GeoMx). Arrow indicates the measurement and direction of Breslow depth. Scale bars 500 μm. **D.** Examples of histopathological features annotated in the specimen MEL14. Top row: Fields of view of a H&E-stained section with four major histologic regions indicated: adjacent normal melanocytes and skin, melanocytic atypia (precursor), melanoma in situ (MIS), vertical growth phase melanoma. Bottom rows: Corresponding CyCIF images showing staining for a variety of tumor and immune markers. See Supplementary Table S3 for how markers related to cell types. Scale bars 100 μm.

FFPE specimens of primary cutaneous melanoma were obtained from the archives of the Brigham and Women’s and Massachusetts General Hospital Dermatopathology Services (n=62 patients; MEL14 to MEL86 see Supplementary Table S1 for HTAN identifiers) (Fig. 1A,B). Two sets of specimens were acquired and deeply annotated: sample set A (n=45) was selected for the presence of multiple disease stages within the same sample with an emphasis on stage I melanoma. Sample set B (n=17) was selected for the presence of later stage II and III disease; multiple stages were often present in these specimens, but this was not a selection criterion. H&E-stained tissue sections were reviewed by a board-certified dermatopathologist to confirm the original diagnosis and evaluate the presence and extent of five histological (progression) features: (i) adjacent normal skin, (ii) melanocytic atypia (precursor fields) (iii) melanoma in situ (MIS; stage 0 melanoma), (iv) radial growth phase (RGP) and (v) vertical growth phase melanoma (VGP, stages I-III) (Fig. 1C,D; Supplementary Fig. S1A). This yielded a total of 313 histological regions of interest (HRs; ranging from 1 to 14 per specimen; average 4) across 62 specimens; these HRs were also annotated for the presence of tumor infiltrating lymphocytes (TILs), and epithelioid, spindle, rhabdoid, and pleomorphic tumor cell morphologies.

We then performed 34 to 49-plex whole-slide CyCIF imaging (Supplementary Fig. S1B) and used histopathological annotations and CyCIF data to guide the selection of microregions (MRs) for GeoMX microregional transcriptomic profiling. These MRs ranged in size from ~100 cells for normal and precursor areas to ~350 cells for RGP and VGP regions (Supplementary Fig. S1C). The MRs were carefully selected and delineated using various shapes according to the histological features (Fig. 1C, Supplementary Fig. S1D), with dimensions similar to the correlation length scale for spatial features, as previously described.^50^ Prior to mRNA preparation, MRs were usually (but not always; see Supplementary Table S2 for MR metadata) separated into tumor and immune regions based on antibody staining (referred to as “segmentation” in the GeoMX workflow) using either a mixture of anti-SOX10 and anti-MART1 antibodies or anti-CD45 antibodies (Supplementary Fig. S1D,E); a total of 1266 MRs were profiled. Archival specimens of primary melanoma can be difficult to manage because they are often friable; thus, not all specimens that generated GeoMX and CyCIF data passed quality control and could be included in the final dataset (Fig. 1B).^51^

To identify protein features associated with melanoma initiation and progression, CyCIF images were segmented using algorithms in MCMICRO^52^ and marker intensities were quantified in ~2 × 10^6^ tumor and ~3 × 10^6^ non-tumor cells. Intensity data were gated on a per specimen basis and patterns of positive and negative staining of 20 cell lineage and functional markers were used to classify cell types, including four major non-immune cell types (melanocytic, keratinocytic, myofibroblast, and endothelial), B cells, five subtypes or functional states of T cells, and five subtypes of myeloid cells (see Supplementary Fig. S1F–I for markers and cell type identification plan). Tumor cells were further subdivided based on the expression of melanocytic lineage markers (e.g., MART1, S100A1), melanoma biomarkers commonly used in clinical practice for diagnosis (e.g., PRAME), differentiation markers (e.g., SOX9, SOX10, NGFR), and proliferation markers (e.g., KI67, pH3; see Supplementary Table S3 for index of gene and protein names).

### Association of melanocytic differentiation with progression and intertumoral heterogeneity

**Figure 2.**
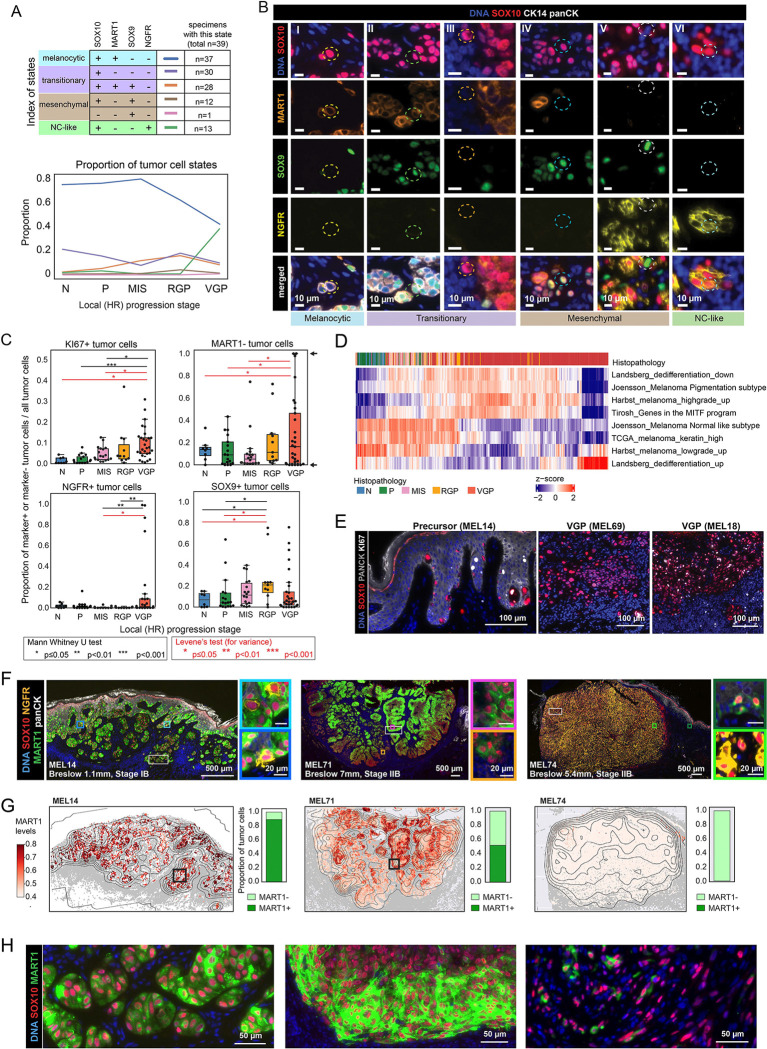
Association of melanocytic differentiation with progression and intertumoral heterogeneity **A.** Tumor cell states annotated according to the binarized staining of protein markers for SOX10, MART1, SOX9 and NGFR (upper panel). The proportion of each tumor cell state within local stages of progression (N, P, MIS, RGP and VGP; lower panel). **B**. CyCIF images showing examples of single cells representing the protein-based tumor cell states detected in the primary melanoma cohort samples. The magnified regions labeled as I-VI from Supplementary Fig. S2B, stained for DNA (blue), SOX10 (red), MART1 (orange), SOX9 (green) and NGFR (yellow). Scale bars, 10 ȝm. **C.** The proportion of SOX10+ melanocytes or tumor cells expressing KI67, MART1, NGFR and SOX9 grouped by histologic regions (HR). MannWhitney U test (black) and Levene’s test (red) *, *P* < 0.05; **, *P* < 0.01; ***, *P* < 0.001. Box represents the first and third interquartiles of the data, whiskers extend to show the rest of the distribution except for points that are determined to be outliers. See Supplementary Table S5 for the CyCIF quantification. **D.** Heatmap showing mean expression (z-scored) of melanoma associated signatures within tumor GeoMX MRs annotated for local stages of progression. **E**. CyCIF images of specimen MEL14, MEL69 and MEL18, showing selected fields of view stained for DNA (blue), SOX10 (red), KI67 (white), PanCK (gray). **F**. CyCIF images of specimens MEL14 (left), MEL71 (middle) and MEL74 (right), stained for DNA (blue), epidermis (panCK: white), and melanocyte/tumor (SOX10: red, NGFR: yellow, MART1: green). Colored rectangles represent magnified regions at right. Scale bars, 500 ȝm and 20 ȝm. **G.** Scatter plots showing MART1 intensity for tumor cell and melanocyte cell centroids for invasive regions from MEL14, MEL71 and MEL74. The centroids for other cell types (non-tumor cells) are shown with grey dots. The proportion of MART1^+^ and MART1^−^ tumor cells (binarized call with gate 0.5) is shown on the right of each scatter plot. **H.** Selected fields of view of CyCIF images from panel F (white rectangles), stained for DNA (blue), SOX10 (red), and MART1 (green). Scale bars, 50 ȝm.

The proportions of melanocytic, transitional, mesenchymal, and neural-crest (NC) like tumor cells in each HR were determined based on patterns of SOX10, MART1, SOX9, and NGFR marker co-expression; this showed that the majority of melanocytes in normal and precursor regions were melanocytic (defined as SOX10^+^ MART1^+^, Fig. 2A–C, Supplementary Fig. S2A,B, Supplementary Table S4). Mesenchymal and neural-crest tumor states (MART1^−^ NGFR^+^ and/or SOX9^+^) emerged only in invasive melanomas, including RGP and VGP regions (Fig. 2A,C). Similar trends were observed for Breslow depth, a measure of the maximal depth of dermal invasion used during clinical staging (Supplementary Fig. S2C). These results are consistent with an extensive histopathology literature based on H&E staining and single channel IHC of primary melanoma^37–39^.

GeoMX transcriptomic profiles from 900 tumor MRs were then compared to published transcriptional signatures derived from scRNA sequencing of metastatic tumors and melanoma cell lines^29,53,54^. These signatures showed a good correspondence with stage (Fig. 2D). For example, the “*Harbst melanoma highgrade*” and “*Joensson_Melanoma Pigmentation subtype*” signatures were enriched in RGP and VGP relative to normal, precursor and MIS stages, whereas the opposite was true of “*Harbst melanoma lowgrade*” and “*Joensson_Melanoma Normal like subtype*”. Thus, local progression in primary melanoma specimens was associated with the emergence of de-differentiated states that are similar to states previously characterized in metastatic disease.

Coincident with the broad trends described above, CyCIF data revealed a high level of specimen-to-specimen differences in the proportions of cells positive for one or more tumor state markers MART1, NGFR, SOX9, PRAME and S100A1 or the KI67^+^ proliferation marker (Fig. 2C, Supplementary Fig. S2D,E). For example, the proportion of proliferative tumor cells (a feature predictive of poor prognosis^55,56^) increased significantly with stage, but a subset of invasive VGP HRs were as nonproliferative in the tumor compartment (2% Ki67^+^) as the average precursor field (Fig. 2C,E). Similarly, the proportion of MART1^−^ cells in VGP HRs varied from 0% to 100%. (Fig. 2C,G). MART1 (MLANA) levels are controlled by the melanocyte master transcription factor MITF^57^ and are widely used in melanoma diagnosis.^58^ Loss of MART1 expression reflects a fundamental change in tumor differentiation status (Supplementary Fig. S2F). The mean proportion of MART1^−^ cells was not significantly associated with progression stage, but increasing intertumoral variability was (as measured by Levene’s test; e.g., the variability of MART1^−^ cells in N HRs was significantly lower than in VGP HRs, Fig. 2C). The proportion of NGFR+ tumor cells showed a similar trend of increasing variance with stage (e.g., MIS to VGP stages; Levene’s test p=0.05, Fig. 2C). This variability was readily apparent in images, both within whole specimens and within individual domains of a single specimen (Fig. 2F–H). Moreover, this type of analysis underestimates the extent to which tumor cells differed from each other since it involves binarizing markers into plus and minus states even though markers such as MART1 exhibited continuous gradation in expression level within and across specimens (Fig. 2G,H, Supplementary Fig. S2G).

### Quantifying intra- and inter-tumor heterogeneity

**Figure 3.**
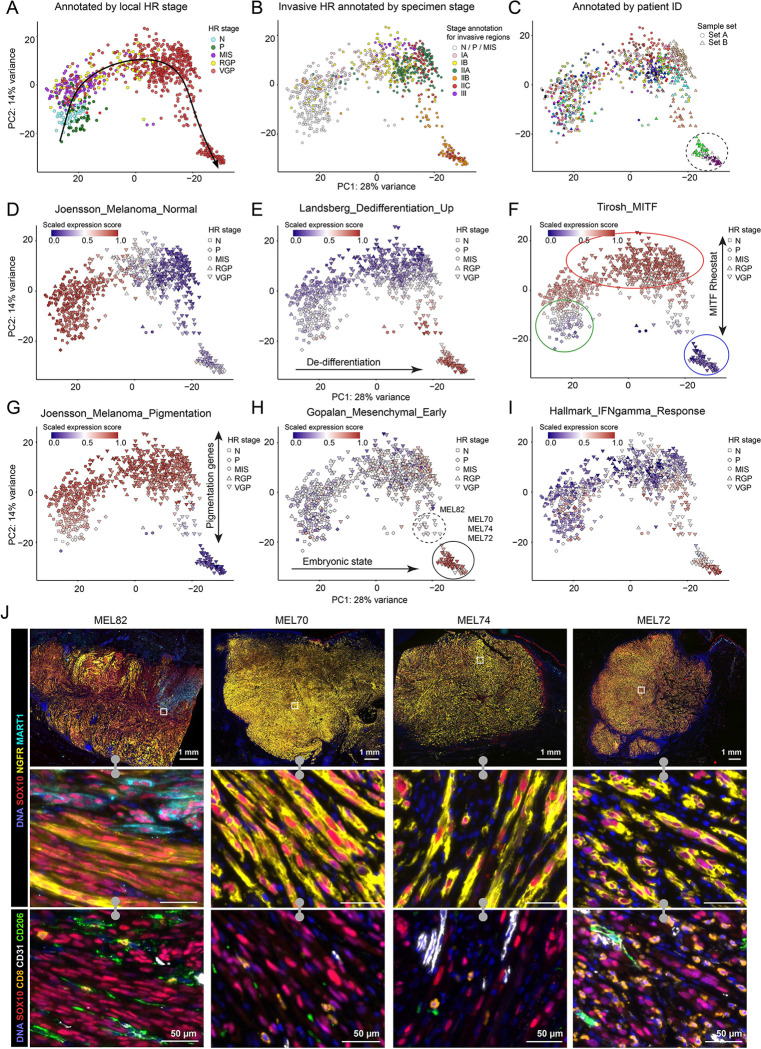
Intra- and inter-tumor transcriptomic heterogeneity in primary melanoma. **A-C.** Principal component (PC) analysis plots of GeoMX spatial transcriptomic data from microregions (MRs) representing melanocytes and tumor cells, with colors indicating histopathological annotations (**A**), specimen stage, AJCC 8^th^ edition (**B**), and patient ID and sample set (**C**). Annotations are shown in Supplementary Fig. S3A and S3B. **D-I**. PC analysis plots of GeoMX spatial transcriptomic data from MRs representing melanocytes and tumor cells, colored by the enrichment scores for previously published melanoma-related and Hallmark gene signatures: *Joensson_Melanoma_Normal* (**D**), *Landsberg_dedifferentiation_up* (**E**), *Tirosh_MITF* (**F**), *Joensson_Melanoma_Pigmentation* (**G**), *Gopalan_Mesenchymal_Early* (**H**), *Hallmark_IFNgamma_response* (**E**). **J.** CyCIF images of specimens MEL82, MEL70, MEL74, and MEL72 stained with a subset of tumor, immune, and stromal markers: DNA (blue), and melanocyte/tumor (SOX10: red, NGFR: yellow, MART1: cyan), CD8^+^ T cells (CD8: orange), endothelial cells (CD31: white), CD206^+^ macrophages (CD206: green). Top row shows low magnification view, with rectangles corresponding to the ROIs magnified in the middle and bottom rows. Scale bars 1 mm and 50 μm.

To quantify molecular programs associated with intertumoral heterogeneity, we analyzed 900 GeoMX tumor and melanocyte MRs using Principal Component Analysis (PCA). This revealed a continuous trajectory in PC1 and PC2 from normal HRs on the left of PC1 to VGP HRs on the right (Fig. 3A–C; Supplementary Fig. S3A–E)^7^. When the PCA landscape was annotated using previously established gene expression signatures (e.g. from Fig. 2D) we found that MRs lying to the left corresponded to normal-like melanoma signatures (“*Joensson_Melanoma Normal”)* and those on the right to dedifferentiated signatures (“*Landsberg_dedifferentiation_up*”, (Fig. 3D,E; Supplementary Fig. S3F,G; Supplementary Fig. S4A–D, Supplementary Fig. S5A–C). Separation along PC2 corresponded to the strength of the MITF signature, consistent with the idea that MITF functions as a molecular rheostat.^15^ MITF-intermediate states were observed in normal HRs (green outline, Fig. 3F,G, Supplementary Fig. S3H), MITF-high (“hyper-pigmented”) states in invasive melanoma (red outline), and MITF-low dedifferentiated neural-crest like states in invasive VGP HRs (blue outline). Gradation in the intensity levels of the MITF target MART1 were also apparent from one microregion to the next (Fig. 1D, Fig. 2H).

During embryogenesis, melanoblasts arise from the neural-crest lineage and migrate to the epidermis where they mature into melanocytes^59^. Embryogenic signatures (*Gopalan_Mesenchymal_Early*)^60^ were strongly enriched to the right of PC1, coincident with neural crest signatures (e.g. “*Rambow_Neural Crest Stem Cell*”) and were most evident in three deeply invasive specimens (MEL70, 72, 74; solid outline in Fig. 3F,H; Supplementary Fig. S3I,J). These microregions were also highly enriched for IFN gamma and alpha response signatures in the tumor compartment (Fig. 3I and Supplementary Fig. S3K), but expression of inflammatory programs was not restricted to invasive microregions, suggesting the presence of inflammation in earlier stages of melanoma as well. MEL70, 72 and 74 also exhibited uniformly high NGFR staining in the invasive tumor compartment with tumor cells arranged in a pattern consistent with “vascular mimicry”, a histological feature associated with malignancy (Fig. 3J)^61^. MRs from these tumors expressed the highest levels of *NGFR* and the lowest levels of *MLANA* and *MITF* of all specimens (Supplementary Fig. S3L–N). We also identified one specimen (MEL82; Fig. 3H dashed outline) in which NGFR^+^ cells were intermixed with NGFR^−^ tumor cells that expressed SOX10, MART1, and/or SOX9 (Fig. 3J). These data demonstrate that, in our cohort, an embryonic (neural crest-like) state is present only in invasive HRs late in primary melanoma progression; we obtained no evidence that an embryonic melanocytic state is present in early-stage disease or is a progenitor for most tumors.

### Spatial patterns of intra-tumoral heterogeneity in early-stage melanoma

**Figure 4.**
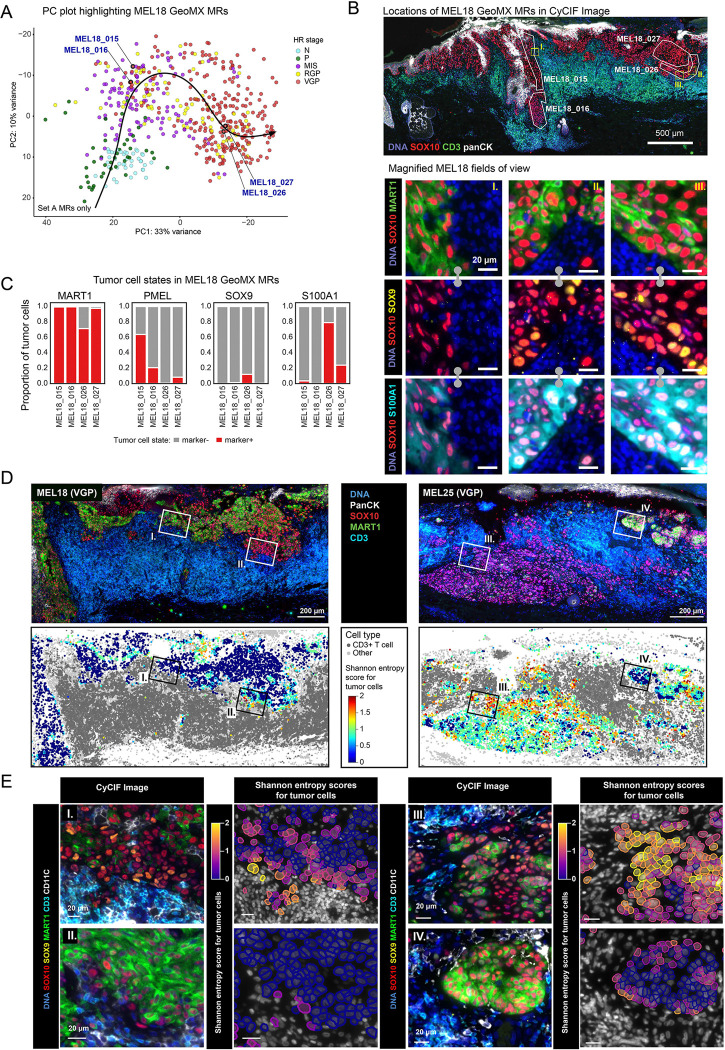
Quantifying tumor cell state heterogeneity with spatial entropy **A.** PC plot of all GeoMX spatial transcriptomic data from microregions (MRs) representing melanocytes and tumor cells for sample Set A, colored by histological annotations. Four exemplary MRs from specimen MEL18 are highlighted (MRs MEL18_015, MEL18_016, MEL18_027, MEL18_026). **B.** Top: CyCIF image of MEL18 labeled with the spatial locations of selected VGP GeoMX microregions (white polygons). MRs are labeled with MR ID, indicating how the regions correspond to the PC plot in panel C. Lower: Magnified ROIs from top panel showing various tumor cell state markers, including SOX10 (red), MART1 (green), SOX9 (yellow). Scale bars, 500 μm and 20 μm. **C.** The proportion of tumor cells positive for MART1, PMEL, SOX9 and S100A1 within selected VGP GeoMX microregions from specimen MEL18. **D.** Top: CyCIF image of specimens MEL18 (top left) and MEL25 (top right) stained for DNA (blue), epidermis (panCK; white), melanocytes (SOX10; red), tumor (MART1; green) and T cells (CD3; cyan). Bottom: Scatter plots show the local neighborhood Shannon entropy values of each SOX10^+^ tumor cell in specimens MEL18 and MEL25, which are mapped back to the corresponding tissue location using the X,Y coordinates of the cell centroids. The boxes labeled as I-IV represent the regions magnified in **E**. Scale bars 200 μm. **E.** Magnified CyCIF ROIs from **D**, stained for DNA (blue), SOX10 (red), MART1 (green), SOX9 (yellow), CD3 (cyan), CD11c (white). The adjacent scatterplots show the local neighborhood Shannon entropy values calculated for each SOX10+ tumor cell within the magnified regions. Scale bars 20 μm.

In contrast to observations in metastatic tumors,^7^ we observed that MRs from many patients were intermixed, except for the invasive regions from the four NGFR-high tumors described above, Fig. 3A–C; Supplementary Fig. S3A,B)^7^. To compare early MRs from a single specimen to those from all specimens of similar stages, we focused on Set A tumors and found that individual MRs from one tumor were often as different from one another as from MRs from different tumors (Fig. 4A, Supplementary Fig. S6A,B). For example, for MEL18 (a stage IIA melanoma), some MRs with RGP and VGP regions mapped to the left in PC1 (MEL18_015 and _016) and others to the right (MEL18_026 and _027; Fig. 4B). The former MRs corresponded to cells growing along the follicular epithelium and invading beyond the follicular adventitia into the surrounding dermis, whereas the latter MRs were located in the dermal invasive component without hair follicle involvement (Fig. 4B). In the case of MEL25 (a stage IA melanoma), MRs corresponding to different invasion depths were spread along PC1 with MRs corresponding to the dermal invasive regions (regions MEL25_59 and _65) distinct from the intraepidermal component with superficial invasion (left regions MEL25_61 and MEL25_62, Supplementary Fig. S6C,D). Differences in transcriptional signatures between MEL18 and MEL25 MRs were readily discernable in the corresponding CyCIF images, for example partial loss of MART1 in MRs mapped to the left along PC1 and retention of MART1 staining in MRs mapped to the right (Fig. 4A–C and Supplementary Fig. S6C–E). These differences were not simply related to the distance between an MR and the dermal-epidermal junction (DEJ), an important consideration since the cellular composition of the skin changes dramatically from epidermis to dermis (Supplementary Fig. S3D).

These data demonstrate that gene expression can vary as greatly within a single primary melanoma as between whole tumors or similar size regions from different tumors. This implies that mesoscale cellular neighborhoods similar in size to MRs (200–600 cells or 2–5% of the area of a tissue section) represent a biologically significant spatial scale for disease-relevant changes in the tumor compartment. However, specimens were not all equivalently heterogeneous: a subset of deeply invasive specimens contained only tumor cells with embryonic neural-crest like states having uniform NGFR staining.

### Quantifying tumor cell state heterogeneity using spatial entropy

Examining CyCIF images from individual MRs showed that cells representing distinct differentiation states not only differed from one MR to the next; they were also frequently intermixed at the single cell level (Fig. 3J). For example, both superficial and deep MRs from MEL18 and MEL25 contained neighboring tumor cells expressing SOX9, SOX10 and MART1 in different combinations and intensities, consistent with frequent changes in molecular programs associated with these melanocyte markers (Fig. 4B,C, Supplementary Fig. S6D,E). We reasoned that the extent of local intermixing might serve as a measure of tumor cell plasticity. We quantified intermixing using local Shannon entropy, with neighbors defined by Delaunay triangulation on tumor cells (ignoring stromal or immune cells), and cell states as defined in Fig. 2A (e.g., SOX10^+^ MART1^+^ SOX9^−^ vs. SOX10^+^ MART1^+^ SOX9^+^ vs. SOX10^+^ MART1^−^ SOX9^−^). A low entropy score corresponded to a region of tissue in which tumor cells were similar in type, while a high entropy score corresponded to a spatially intermixed state. Using this approach, we found that some specimens (e.g., MEL18) exhibited low overall entropy whereas others (e.g., MEL25) exhibited high entropy (Fig. 4D). However, any single specimen could have both high and low local entropy domains, as evidenced by variability in entropy maps at both the whole-specimen and local levels (Fig. 4D,E).

### The variance of spatial entropy increases with melanoma progression

**Figure 5.**
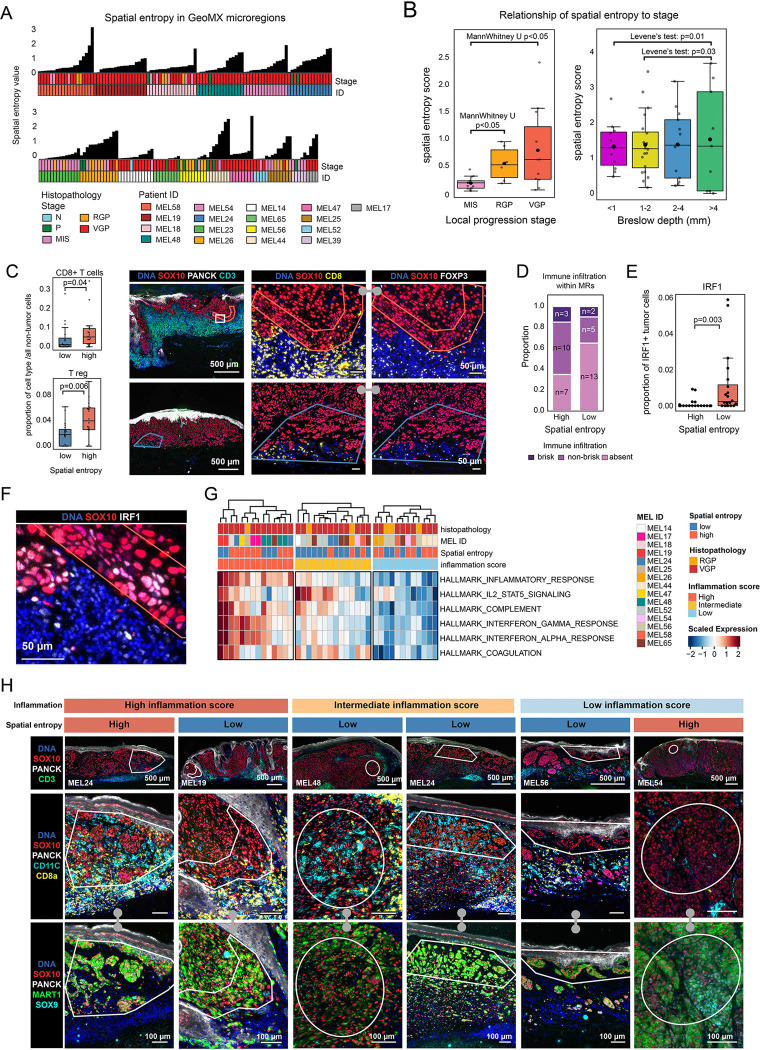
Spatial entropy and inflammation. **A.** CoMut plot of the spatial entropy scores for each GeoMX microregion (MRs), calculated using CyCIF data of the adjacent tissue section. Each MR is annotated for histopathology (N, P, MIS, RGP, VGP) and patient ID. **B.** Spatial entropy scores calculated by local progression stages (left) and Breslow depth (right; for RGP and VGP regions only). Box represents the first and third interquartiles of the data and whiskers extend to show the rest of the distribution except for points that are determined to be outliers. The p-value of local progression stage comparison was calculated using MannWhitney U test. The p-value of Breslow depth comparison was calculated based on Levene’s test. **C.** Left: The proportion of CD8^+^ T cells (top) and T regs (bottom) out of all non-tumor cells within high or low entropy microregions. Box represents the first and third interquartiles of the data, whiskers extend to show the rest of the distribution except for points that were determined to be outliers. Right: CyCIF images of samples MEL18 (top), and MEL65 (bottom), stained for DNA (blue), panCK (white) and SOX10 (red), CD3 (cyan). The magnified box in specimen MEL18 indicates a microregion with high spatial entropy, and in specimen MEL65 indicates microregion with low spatial entropy. These regions are magnified next to the whole-slide images and stained for DNA (blue), SOX10 (red), CD8 (yellow) and FOXP3 (white). Scale bars, 500 μm and 50 μm. **D.** Stacked bar plot showing the distribution of annotations for immune cell infiltration as evaluated by CD45 staining for GeoMX MRs. The proportion of various immune cell infiltration patterns is shown for MRs with high and low spatial entropy. The p-value was calculated using Chi-square test. **E.** Boxplot showing the proportion of IRF1^+^ tumor cells within high and low entropy MRs. The p-value was calculated using MannWhitney U test. **F.** Magnified field of view of a high spatial entropy MR shown in panel **C** (specimen MEL18), stained for DNA (blue), IRF1 (white) and SOX10 (red). Scale bar, 50 μm. **G.** The enrichment scores of Hallmark gene set (pathways) for spatial entropy regions (n = 40), annotated by histopathology (RGP, VGP), spatial entropy score grouping (high, low), patient ID, and inflammation score (high, intermediate, low). **H.** Top row from left to right: CyCIF images of samples MEL24, MEL19, MEL48, MEL24, MEL56 and MEL54, stained for DNA (blue), panCK (white), SOX10 (red), and CD3 (green). White regions indicate individual exemplary GeoMX MRs annotated for inflammation score and spatial entropy (annotations shown on top of each column). These regions are magnified below the low power fields of view and stained for DNA (blue), SOX10 (red), MART1 (green), CD3 (yellow) and CD11C (cyan; middle row); DNA (blue), SOX10 (red), SOX9 (cyan), and MART1 (green; bottom row). Scale bars, 500 μm and 100 μm.

To identify factors associated with variation in entropy, we compared local histology, gene expression, and entropy at the smallest scale at which they could be computed - a single MR. This required the use of MRs and HRs rather than calculating entropy at the cell-level (the “spatial entropy” see Methods; Fig. 5A,B), and showed that spatial entropy increased significantly with histological stage (e.g., from MIS to RGP or MIS to VGP). When mean entropy values for all HRs in a specimen were compared to Breslow depth for that tumor, we found that the average spatial entropy value was constant from thinner to thicker specimens but the variance increased significantly (as measured by Levene’s test; Fig. 5B). Since Breslow depth is a specimen-level measurement, we also divided specimens into bands perpendicular to the direction of invasion. In this case, we did not observe a consistent or significant association between local depth of invasion and spatial entropy (Supplementary Fig. S7A–C): in many specimens the spatial entropy value was constant with depth, whereas in others it decreased (e.g., MEL18) or increased with depth (MEL58; Supplementary Fig. S7B). Thus, it was not the position of cells within a single tumor, as approximated by depth of invasion, that determined the degree of entropy, but rather the overall thickness of the specimen (the Breslow depth), which is a measure of progression. These data demonstrate that the degree of intermixing of tumor cell lineages (the spatial entropy) increases from MIS to VGP stages and the variability in the entropy value also increases as specimens get thicker overall (Fig. 5B). These conclusions are consistent with visual inspection of tumor states and their distribution within the specimens. As noted above, NGFR^+^ (set B) locally advanced specimens with Breslow depth >4mm were an outlier in this analysis, and had the lowest entropy of all specimens in the cohort; whether this represents a general evolution of tumor state to greater homogeneity or is a feature of a minority of specimens is not yet known (Fig. 5B, Supplementary Fig. S7D–F).

### High spatial heterogeneity associated with immune reaction

Host immune response has previously been shown to promote lineage plasticity in advanced melanoma^29^ and we therefore asked whether spatial entropy at the level of MRs was associated with proximity to an inflammatory environment. First, we used CyCIF to determine the immune cell composition of MRs having high (n=20) and low (n=20) spatial entropy in the tumor compartment. We found that high entropy regions contained significantly higher proportions of CD8^+^ T cells and CD4^+^ FOXP3^+^ regulatory T cells than low entropy regions (Fig. 5C, Supplementary Fig. S7G) and were qualitatively more immune infiltrated, as judged visually using criteria similar to how brisk TIL infiltrates are scored clinically^62^ (Fig. 5D, Supplementary Fig. S7H). Tumor cells in high entropy MRs were also significantly more likely to have nuclear IRF1 (interferon regulatory transcription factor 1), suggesting that they were exposed to interferon-γ (IFNγ; Fig. 5E,F). Based on gene set enrichment (ssGSEA), high entropy MRs were associated with inflammatory signatures, such as responses to IFNα and IFNγ, classic inflammatory cytokines, the complement pathway, a component of innate immunity, and coagulation (a hallmark that exhibits extensive cross-talk with immune and inflammatory genes; the gene sets for the coagulation and complement pathways contain cytokines and chemokines or their receptors;^63^ Fig. 5G,H). We conclude that high entropy in the tumor compartment – and its correlate, tumor cell plasticity – is significantly associated with proximity to immune cells and responsiveness to inflammatory cytokines.

However, at the level of individual MRs, spatial entropy and local immune reaction were not obligatorily linked: some MRs with high spatial entropy did not exhibit upregulation of inflammatory gene signatures, and were not proximate to immune cell populations; the converse was also true (Fig. 5G,H). This lack of one-to-one association was also evident in maps of Shannon entropy at single cell resolution: some regions with abundant T cells in close proximity to tumor cells were homogenous and exhibited low Shannon entropy values at single-cell level (Fig. 4D,E). Thus, while inflammation can cause spatial entropy, other (unmeasured factors) must be additional contributors.

### Progression programs in early-stage melanoma

**Figure 6.**
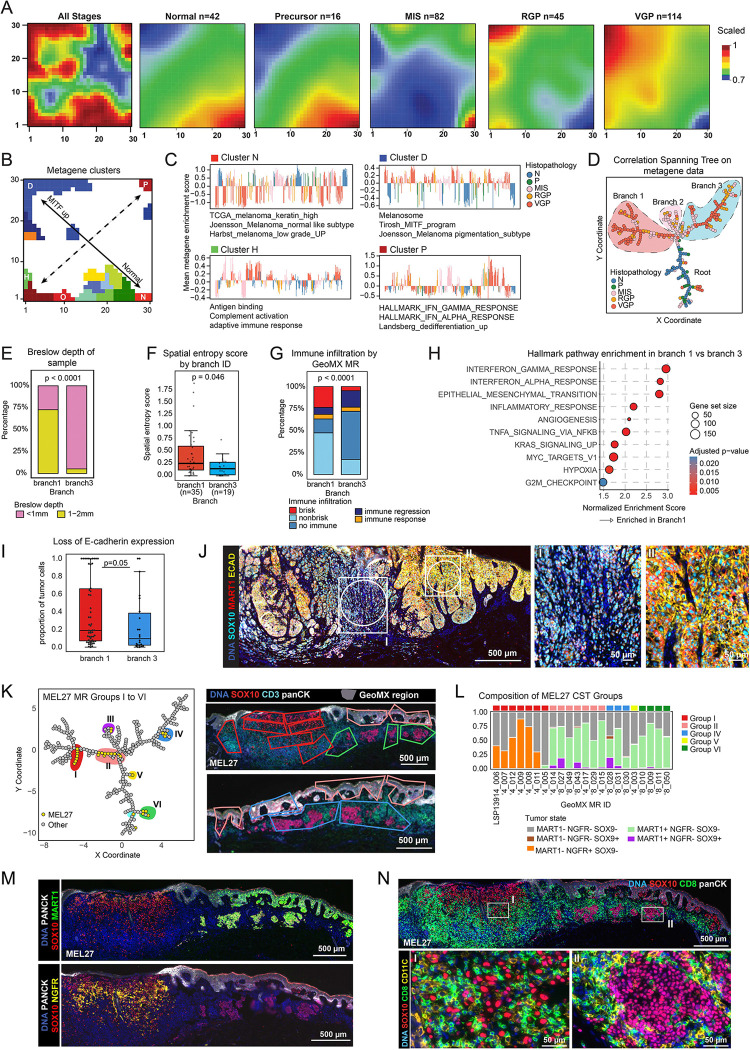
Developmental trajectory from normal melanocytes to invasive melanoma. **A.** Self-organizing map (SOM) portrait of GeoMX data (Set A1, n = 299 from n = 28 patients), showing the global expression of 900 metagenes across profiled samples (left). SOM portraits for local progression stages show the global expression of 900 metagenes in microregions (MRs) grouped by histopathology. The expression levels of each metagene are indicated with a color (Z-score) as compared to the average expression of all metagenes. **B.** SOM clusters A-Q and their mapping to the SOM portrait. Spots N, D, H, P and O are highlighted, and the rest are shown in Supplementary Fig. S8F. **C.** Mean expression profiles of the defined SOM clusters N, D, H and P within microregions from various progression stage categories. The Y axis represents the mean expression profile of the SOM cluster signatures of each microregion. Individual MRs are shown in the X axis. The pathways listed below represent the most enriched pathways within each SOM cluster. **D.** Minimum spanning tree analysis showing the correlation of profiled GeoMX MRs. The minimum spanning tree is annotated by the local progression stage for each microregion and the branch identity (Branches 1, 2 and 3). **E.** The percentage of microregions in Branches 1 and 3 belonging to samples with Breslow depth <1mm and 1–2 mm. P-value was calculated based on Fisher’s exact test. **F.** Spatial entropy scores calculated for the corresponding tumor regions in the adjacent tissue section for Branch 1 and 3 MRs. P-value was calculated based on Wilcoxon Rank Sum test. **G.** The proportion of annotations for immune responses (brisk TIL, non-brisk TIL, no immune, immune regression, immune response) within Branch 1 and 3 MRs. P-value was calculated based on Fisher’s exact test. **H.** Gene-set enrichment analysis (GSEA) of Hallmark gene set (pathways) significantly enriched in Branch 1 and Branch 3 microregions. Dot size indicates the size of the gene set and color indicates the adjusted *p* value. **I.** The proportion of tumor cells staining positive for E-cadherin of all tumor cells in Branch 1 and Branch 3 microregions. **J.** CyCIF image of specimen MEL54 stained for DNA (blue), SOX10 (cyan), MART1 (red), E-cadherin (yellow), highlighting two GeoMX microregions from branch 1. Scale bars, 500 μm and 50 μm. **K.** Left: Correlation spanning tree highlighting microregions from specimen MEL27 (yellow dots). Groups of microregions are encircled with different colors and annotated with roman numerals (I-VI). A subset of these microregions is highlighted in the CyCIF images on the right. **L.** The proportion of various tumor cell states within selected VGP GeoMX microregions from specimen MEL27. Cluster ID indicates the grouping of MRs shown in the correlation spanning tree in panel K. **M.** CyCIF image of specimen MEL27 stained for DNA (blue), SOX10 (red), panCK (white), MART1 (green; top row), and NGFR (yellow; bottom row). **N.** Top: CyCIF image of specimen MEL27 stained for DNA (blue), SOX10 (red), panCK (white) and CD8 (green). Bottom: magnified regions indicated with I and II are stained for DNA (blue), SOX10 (red), CD8 (green) and CD11C (yellow).

To identify additional features promoting dedifferentiation and intra-tumoral heterogeneity, we used machine learning based on self-organizing maps (SOMs); this approach projects high-dimensional gene expression onto a two-dimensional grid^47^. We concentrated on earlier stage disease (Set A) and modeled the two batches of GeoMX signatures separately since SOMs were more sensitive to batch effects than PCA analysis (n = 299 MRs for Set A1 and n = 239 for Set A2); this yielded 900 metagenes (sets of genes with coordinated expression profiles). We then mapped the metagenes onto a two-dimensional representation of the SOM (the SOM portrait) based on expression pattern and decomposed portraits for each local progression stage. This revealed a well-organized progression series: MRs associated with normal and precursor stages projected onto the lower right corner of the portrait, RGP and VGP stages projected onto the upper left corner and MIS stages exhibited intermediate projection (Fig. 6A and Supplementary Fig. S8A,B). Thus, the SOM had learned the progression axis from the original data in a fully unsupervised manner.

To identify transcriptional features contributing to the SOM portrait, metagenes were clustered based on a min-max scaled threshold of 0.95, corresponding approximately to the top 5% of expression value in each metagene profile; this yielded 17 clusters of co-expressed metagenes. These clusters represent key discriminating features in the data and as expected, mapped primarily to the edges of the SOM portrait (Clusters A-Q; Fig. 6B,C; Supplementary Fig. S8C,D). Cluster N, which existed in the lower right region of the portrait associated with normal and precursor MRs, was enriched in gene expression signatures such as “*Joensson_Melanoma_normal like subtype”* (see Fig. 2 and 3). Cluster D in the upper left of the portrait, the location of RGP and VGP MRs, was enriched in signatures of MITF upregulation (e.g., “*Tirosh_MITF_program*, Fig. 6C). Moreover, when the clusters were projected back to the PC landscape, enrichment scores for clusters N and D corresponded well to the progression trajectory along PC1 (Supplementary Fig. S8E). The other diagonal of the SOM portrait (dotted line in Fig. 6B) was organized by differences in immune and inflammatory signatures, with IFNγ and IFNα signatures in the upper right (cluster P) and immune features in the lower left (cluster H; Fig. 6B,C; see Supplementary Fig. S8F for data on all clusters). Dedifferentiation signatures were not a major organizing principle driving SOM organization (e.g. *Landsberg_dedifferentiation_up*), probably because dataset A1 was enriched in earlier stage specimens with fewer NGFR^+^ tumors (the portion of the PCA plot depicted in Supplementary Fig. S3A,C).

To identify the information learned by the SOM we calculated the pairwise correlation among metagenes (Pearson’s correlation coefficient, Supplementary Fig. S8G, Supplementary Fig. S9A) and then generated a correlation spanning tree (CST, Fig. 6D, Supplementary Fig. S9B). A CST represents a cluster map as a partitioned tree, generating a representation that is easier to parse but does not reduce the fidelity of clustering.^64^ We interpreted the resulting CST as having a root and three branches, with the root comprising primarily normal and precursor regions (Fig. 6D microregions in blue and green), branch 2 comprising MIS regions, and branches 1 and 3 comprising VGP and RGP. Comparing the invasive branches, we found that branch 1 was enriched in MRs from tumors with Breslow depth >1 mm (Fig. 6E) and corresponded to clusters D and P of the SOM portrait (Supplementary Fig. S9C). MRs in branch 1 had higher spatial entropy (Fig. 6F and Supplementary Fig. S9D and greater immune-association than MRs from the thinner and more melanocytic (MITF-high) MRs in branch 3 (Fig. 6G). Enrichment analysis confirmed that branch 1 was significantly enriched in IFNγ, IFNα, and TNFα responses, and in epithelial-mesenchymal transition (EMT signatures, Fig. 6H). To confirm this finding, we compared E-cadherin staining in Branch 1 and 3 MRs. E-cadherin is a cell adhesion protein and tumor suppressor, whose loss is a hallmark of EMT. We observed a significantly higher proportion of E-cadherin negative cells in branch 1 than branch 3 MRs (Fig. 6I,J), and overall, E-cadherin intensity levels were positively correlated with MART1 (Supplementary Fig. S9E). Thus, tumor states that were intermixed in PCA analysis are revealed by SOMs to comprise two (and possibly more) distinct trajectories.

When MRs making up the CST were labelled by specimen identity, most specimens were found to contain MRs that mapped to multiple branches (Supplementary Fig. S9F), consistent with the high intratumoral heterogeneity observed visually and by PCA analysis (Fig. 3). For example, stage IB specimen MEL27 formed 6 groups based on their position in the CST (labelled I to VI; Fig. 6K, Supplementary Fig. S9G). MRs in group I were enriched in MART1^−^ NGFR^+^ tumor cells (and a brisk CD8+ T cell infiltrate was present), while group II MRs contained MART1^+^ NGFR^−^ cells (Fig. 6L–N). MRs in groups II, IV and VI represented the superficial or intraepidermal component of invasive melanoma (Fig. 6K). In contrast, MEL14 was an exemplar of a tumor in which all tumor MRs clustered closely together in Branch 1 (Supplementary Fig. S10A). These MRs were also similar to each other with respect to tumor cell states, with 92% of tumor cells being MART1^+^ PMEL^+^ (Supplementary Fig. S10B,C). This unsupervised analysis confirms our previous conclusion that MRs from a single tumor can be as variable as MRs from the entire cohort but that high intra-tumoral heterogeneity is not an obligatory feature of primary melanoma.

### Spatial features of tumor microenvironment (TME) in melanoma dedifferentiation

**Figure 7.**
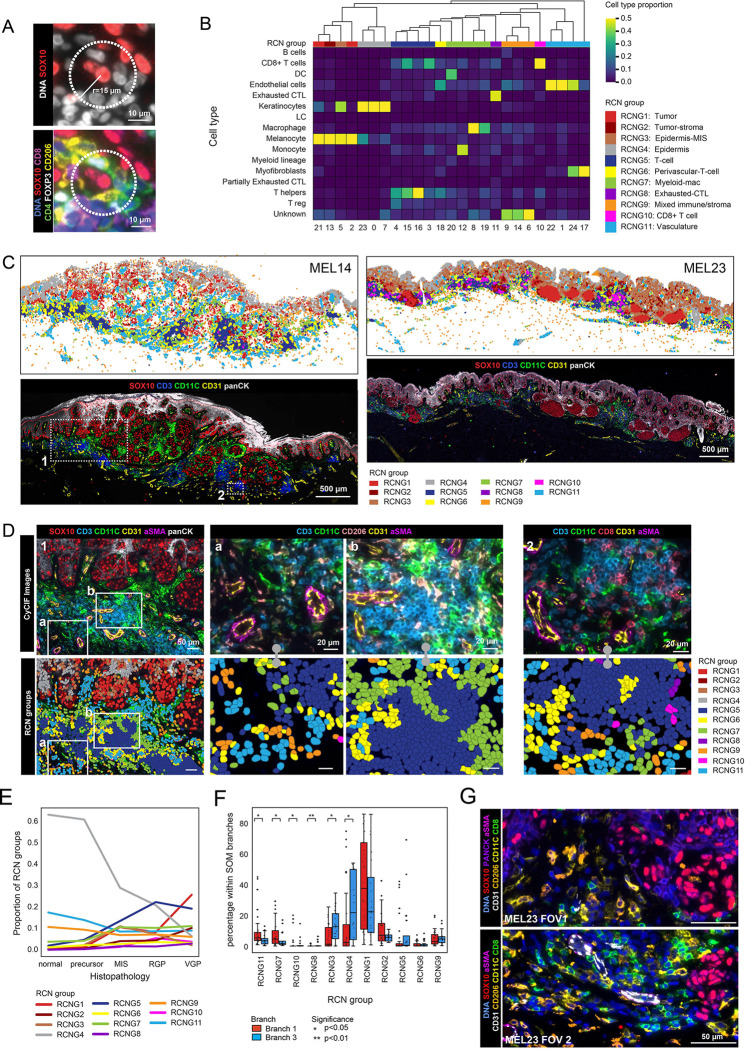
Spatial features of tumor microenvironment (TME) in melanoma dedifferentiation **A.** CyCIF image of a field of view in specimen MEL18 showing an example of a cellular neighborhood with a 15 μm (50 px) radius, stained for DNA (blue), SOX10 (red; left). The corresponding region (right) stained for DNA (blue), SOX10 (red), CD8 (purple), CD4 (green), FOXP3 (white), CD206 (yellow). Scale bars 10 μm. **B.** A heatmap showing the mean proportion of cell types within Recurrent Cellular Neighborhoods (k=25) and their grouping into 11 meta clusters (RCN groups: RCNG). **C.** Top: Scatter plots showing the RCN annotations for each cell within selected fields of view for samples MEL14 (left) and MEL23 (right). The corresponding CyCIF image is shown below for both tumors, stained for SOX10 (red), CD3 (blue), CD11C (green), panCK (white), CD31 (yellow). Scale bars 500 μm. **D.** Magnified regions 1 and 2 from specimen MEL14 shown in panel C. Top left: Region 1 stained for SOX10 (red), CD3 (blue), CD11C (green), panCK (white), CD31 (yellow), aSMA (magenta). Bottom left: Region 1 with RNCG annotations for each single cell. Middle: magnified insets a and b from Region 1 stained for CD3 (blue), CD11C (green), CD206 (orange), CD31 (yellow), aSMA (magenta), and their corresponding RCNG annotations. Top right: Region 2 stained for CD3 (blue), CD11C (green), CD8 (red), CD31 (yellow), and aSMA (magenta). Bottom right: Region 2 with RNCG annotations for each single cell. Scale bars, 50 μm and 20 μm. **E.** The proportion of RCN groups (RCNG1–11) within local progression stage HRs (normal, precursor, MIS, RGP, VGP). **F.** RCN identities of single cells belonging to Branch 1 and Branch 3 microregions. Box plots show the proportion of cells within Branch 1 and Branch 3 belonging to different RCNs in each CyCIF sample. *, P < 0.05; **, P < 0.01. **G.** Magnified fields of view from specimen MEL23 (CyCIF image shown in Supplementary Fig. S11B). Staining for DNA (blue), SOX10 (red), panCK (purple; top panel only), aSMA (magenta), CD31 (white), CD206 (orange), CD11C (yellow), and CD8 (green) are shown. Scale bars 50 μm.

To identify additional features distinguishing CST branches 1 and 3, we used K-means clustering (*k*=25) to generate recurrent spatial cellular neighborhoods (RCNs), which separate intermixed cell types into recurrent communities based on local proximity and relative enrichment (Fig. 7A,B). The RCNs were then clustered into groups (RCNGs; N=11) that corresponded to (i) components of the skin such as epidermis, stroma, or vasculature, (ii) immune domains of different types, (iii) tumor domains, and the (iv) tumor-stromal interface (Fig. 7B–D). The abundance of epidermis RCNGs (RCNG4) decreased by 10-fold from precursor to VGP while T cell and tumor RCNGs (RCNGs 5 and 1) increased by ~5 - and 25-fold, respectively. In contrast, RCNGs for myeloid/macrophage (RCNG7) and vascular (RCNG11) varied less than 3-fold (Fig. 7E). However, the proportions of RCNG7 and RCNG11 were significantly more abundant in the MRs in branch 1 than 3 (Fig. 7F,G, Supplementary Fig. S11A,B). RCNG7 corresponded to a, myeloid-rich compartment located either near the invasive front or instead within the dermis as patches alternating with T cells in neighborhood RCNG5. RCNG11 corresponded to blood vessels, visible in Fig. 7D in both longitudinal and transverse aspect. To control for the possibility that depth-dependent differences in skin composition might confound this analysis, we tested for differences in RCN prevalence with Breslow depth; however, no significant associations were observed (Supplementary Fig. S11C). Enrichment of Branch 1 MRs for proximity to RCNG11 (myeloid cells) but not RCNG 5 (T cells) implies that T cells and myeloid cells were not highly intermixed in most specimens and could therefore differentially pattern the TME. Consistent with this observation, proximity analysis showed that CD8^+^ T cells were on average the nearest neighbor immune cell type for other CD8^+^ T cells and macrophages were the closest neighbors to other macrophages (Supplementary Fig. S11D). Moreover, Branch 1 MRs were found (by CyCIF) to contain a higher proportion of CD163^+^ and/or CD206^+^ macrophages and CD31^+^ endothelial cells than branch 3 MRs (Supplementary Fig. S12A). We conclude that proximity to myeloid cells was one factor distinguishing branch 1 & 3 cells and that proximity to a vascular/perivasculature environment, an established negative prognostic feature in primary melanoma^65^, is another.

As a final way to dissect intratumoral heterogeneity, we returned to factors promoting loss of MART1 expression, which is a univariate measure of tumor cells’ de-differentiation and was among the most variable of all tumor markers. We used comparative proximity analysis to determine the identities of cells lying within a 15 μm radius of each SOX10^+^ MART1^−^ and SOX10^+^ MART1^+^ tumor cell. This revealed a significant association between loss of MART1 expression and CD8^+^ T cells, CD4^+^ Treg cells, macrophages, and endothelial cells, consistent with the more systematic analysis described above (Supplementary Fig. S12B,C).

## DISCUSSION

In this paper, we characterize connections between melanoma initiation and progression scored histologically and cancer cell phenotypes such as plasticity and de-differentiation identified using a combination of highly multiplexed tissue imaging and spatial transcriptomics.^10^ This has provided deeper understanding of factors driving tumor cell phenotypes and should - in the longer term – improve approaches to melanoma staging and prognostication using existing markers such as SOX10, S100A1, MART1 and PRAME. The 313 individually annotated histological regions from 62 patients used in this study spanned adjacent normal skin, precursor fields, melanoma in situ (Stage 0 melanoma), locally invasive melanoma and the cutaneous component of locally metastatic (Stage III) disease – with up to five progression stages in each specimen. We find that primary melanomas of all stages exhibit a very high level of inter-tumor variability when assayed using traditional diagnostic markers or high-plex spatial approaches. These findings recapitulate a large IHC literature on prognostic and diagnostic biomarkers in melanoma^66^ showing that no single protein marker yet discovered exhibits consistent changes with stage.

In contrast, when tumors are subdivided into microregions of 200–600 cells based on local histology and analyzed by spatial transcriptomics, we observe a coherent pattern of progression from epidermal signatures in regions of adjacent normal skin to neural crest-and embryonic signatures in regions of deeper invasion. These data are consistent with prior evidence that melanomas become increasingly dedifferentiated and revert to an embryonic neural-crest like state as they progress^7,60^. The strength of MITF signatures and expression levels of MITF target genes such as *MLANA* (MART1) was found to vary systematically across the dataset, being intermediate in normal skin and precursor regions, elevated in many RGP and VGP domains (which also over-expressed pigmentation genes), and lowest in VGP domains having neural crest signatures. This finding is consistent with the rheostat model of MITF developed from scRNA-Seq data on metastatic tumors and melanoma cell lines; it postulates that MITF has intermediate activity in normal melanocytes, is over-activated in hyperpigmented melanomas cells and is repressed in de-differentiated melanomas^67^. Our data shows that these different activity states are already present in nearly all primary melanomas.

### Differentiation and plasticity are local (neighborhood) tumor features in primary melanoma

The seemingly contradictory observation that tumors can differ dramatically from each other but microregions from these tumors form a coherent progression trajectory is explained by the fact that MRs from a single tumor differ as dramatically from each other as MRs from different tumors. This is true for features as diverse as marker expression, inferred of MITF activity, degree of dedifferentiation and expression of inflammatory programs. This is observed using both simple statistical methods such as PCA, and machine learning methods such as SOMs and CSTs. We therefore conclude that dedifferentiation and other common features of primary melanoma are local (neighborhood) rather than whole-tumor properties. Dedifferentiation in primary melanoma MRs is also associated with local intermixing of tumor cell phenotypes, as measured by Shannon entropy. It is not currently feasible to measure the clonality of primary melanomas at this level of detail, but the recurrence of intermixed differentiated and dedifferentiated tumor states strongly suggests that high entropy is a reflection of tumor cell plasticity.

This raises the question: what factors in the TME cause tumor cell neighborhoods (and individual cells) to switch state? One factor we identify is proximity of tumor cells to immune cells and the resulting induction of inflammatory programs in tumor cells^10^; in metastatic cell lines it has been shown that plasticity^68^ and dedifferentiation^30^ can be induced by an inflammatory environment. Spatial decomposition of tumors into recurrent cell neighborhoods shows that immune cells are unevenly distributed across specimens in a manner concordant with diagnostic distinctions between brisk, non-brisk and absent TILS.^69^ RCNs enriched in T cells and myeloid cells are also distinct from each other in our specimens, with differing spatial distributions; moreover, decomposition of tumor cell transcriptional profiles using SOMs reveals systematic differences in tumor neighborhoods near myeloid or T cell-enriched RCNs with proximity to myeloid RCNs associated with a more aggressive and “mesenchymal” state (macrophage-derived TNFα is known to impact resistance to RAF/MEK inhibitors)^70^. Dedifferentiation and loss of cell adhesion proteins such as E cadherin is also associated with proximity to the vasculature, consistent with the role of angiotropism and embryonic programs in promoting melanoma invasiveness and metastasis^71^.

Many models of tumorigenesis postulate that embryonic states resembling self-renewing stem cells arise early in disease and promote the development of a diversity of downstream states in response to pressure from immunosurveillance or therapy.^72^ However, we find that dedifferentiated states are present in the invasive components of later stage melanoma and very rarely in MIS or stage I melanoma; this is true whether induction of neural crest signatures, NGFR protein expression, or expression of embryonic genes is used as a criterion. Interestingly, the least diverse specimens in our cohort are deeply invasive melanomas of stage IIb-c. These occupied the extremum of the PCA state landscape and were uniformly populated by NGFR-high tumor cells with a vascular mimicry phenotype. By multiple criteria, these cells were more dedifferentiated than the cutaneous component of Stage III (locally metastatic) tumors. Clinically, deep primary tumors are strongly associated with worse outcomes, with Stage IIB/C disease (primary > 4mm depth invasion) having worse prognosis than shallower Stage IIIA tumors in which the cancer has metastasized to lymph nodes.^4^ One possibility is that deep tumors undergo purifying selection as they progress. Alternatively, uniformly NGFR-high tumors might have been “born bad” and progressed rapidly to deep invasion. Analysis of more specimens stratified by recurrence will be necessary to distinguish these possibilities.

### Factors patterning the melanoma TME and tumor compartment

From this study, a picture emerges of tumors patterned by proximity to features of the stroma and by the tissue resident and recruited immune system, all of which are strongly spatially patterned^10^. Stromal features impacting tumor state include blood vessels, which are spaced every 100–200 um in the epidermis, generating micro domains that can be predominantly arteriolar, venular, or devoid of microvasculature^73^. Additional features we have not yet assessed include proximity to nociceptor neurons, which are known to influence immunosurveillance,^74^ and follicles, which may restrict melanoma spread.^75^ A contribution of the current study is to show that TME patterning is associated with (and may be the primary determinant of) the most widely studied molecular programs and features of melanoma cells, such as plasticity, dedifferentiation and loss of cell-cell adhesion via EMT. Many of these states have been previously been described as therapy-induced, but our data suggest that they are already present at the earliest stages of disease.

None of the significant associations we detect between the TME and tumor cell state are absolute: for example, tumor cell neighborhoods can be dedifferentiated and plastic without evidence of proximity to immune cells and the converse is also true. This suggest that many regulatory interactions remain to be identified. Moreover, there is no reason to believe that we have optimally sized the MRs in this study. Although identified based on local morphology, MRs still contained cells of multiple types. It nonetheless seems likely that extending the current approach to more specimens, additional features of the stroma, 3D maps of nerves and the vasculature, and more refined transcriptional profiling will generate increasingly accurate maps of primary melanoma and the features that make a subset of specimens of greatest risk of recurrence.

### Diagnostic implications

A general consensus exists that prognostication for primary melanoma is urgently needed to better discriminate low-risk tumors that do not require aggressive treatment or frequent surveillance from those for which systemic therapy and close monitoring is necessary^76^. This has caused multiple research groups and companies^77^ to develop prognostic tests that combine clinicopathological features of disease with bulk gene expression profiling (e.g. CP-GEP^78^, Skyline DX^®79^. DecisionDX^®80^ etc.) or multiplex IHC (immunoprint^81^). Use of these tests remains controversial however^82^, and American Association of Dermatology guidelines do not recommend molecular testing (outside of clinical trials) due to insufficient evidence of efficacy^83^. The data in this paper may have bearing on this controversy. We find that gene and protein expression signatures are highly variable within a single tumor; if a risk feature in a bulk assay is dominant, such as overexpression of a gene, then this will affect the sensitivity of the assay to a variable degree. If a feature is recessive (e.g., a reduction in expression) then it may be very difficult to reliably detect in a heterogenous tumor. Approaches to overcome spatial heterogeneity, such as multiplexed imaging combined with machine learning on H&E images^5^, may represent a superior approach.

## METHODS

### Clinical samples

Using medical records and pathologic review of H&E-stained diagnostic specimens, we retrospectively identified 62 patients with tissue samples containing various stages of melanoma progression (Supplementary Table S1). The samples were retrieved from the archives of the Department of Pathology at Brigham and Women’s Hospital and collected under Institutional Review Board approval (Primary IRB:2019P003828, Secondary IRB: 21–0656) under a waiver of consent. Fresh FFPE tissue sections were cut from each tumor block. The first section of each block was H&E-stained and used to annotate histopathological ROIs (Fig. 1C and Supplementary Fig. S1B). The remaining subsequent sections were used for cyclic multiplex immunofluorescence imaging (CyCIF) experiments to characterize markers of melanoma progression and the features of the immune microenvironment within various stages of melanoma. A fresh cut of the samples was performed for deeper profiling with microregion transcriptomic profiling using GeoMx Digital Spatial Profiling (DSP) technology. The clinical, biospecimen, and imaging-level metadata were all collected following the Minimum Information about Highly Multiplexed Tissue Imaging (MITI) standards^85^.

Based on the melanoma diagnostic criteria, the histopathologic annotations included normal skin (N), Precursor (P), Melanoma *in situ* (MIS), Radial Growth Phase melanoma (RGP), and Vertical Growth Phase melanoma (VGP). These ROIs were further classified and subdivided based on the presence of immune infiltrate (brisk TIL, non-brisk TIL, absent). In the case that a single specimen contained more than one histologic region in each category (e.g., precursor regions on both sides of vertical growth phase melanoma), we performed neighborhood analyses separately, as these regions were not physically adjacent.

### Imaging (H&E and tissue-based CyCIF)

H&E-stained FFPE sections from each tissue block were imaged using a CyteFinder slide scanning fluorescence microscope (RareCyte Inc.) with a 20×/0.75 NA objective with no pixel binning. Serial FFPE sections (5 μm thick) were subjected to whole-slide, subcellular-resolution, 34 to 49-plex CyCIF imaging with two different antibody panels to generate complementary datasets (Fig. 1C and Supplementary Fig. S1A,B). CyCIF panels 1 and 3 were used for general immune phenotyping to fully profile the tumor microenvironment, and panel 2 was used to better understand the tumor intrinsic features. Antibody information and associated RRIDs are listed in Supplementary Table S1. CyCIF was performed as described in^86^ and more specifically at protocols.io (dx.doi.org/10.17504/protocols.io.j8nlkoqbdv5r/v1). In brief, the BOND RX Automated IHC Stainer was used to bake FFPE slides at 60°C for 30 minutes, dewax using Bond Dewax solution at 72°C, and perform antigen retrieval using Epitope Retrieval 1 (Leica) solution at 100°C for 20 minutes. Slides underwent multiple cycles of antibody incubation, imaging, and fluorophore inactivation. Antibodies were incubated overnight at 4°C in the dark. Before imaging, a 0.15mm single-sided self-adhesive spacer was applied to the slide, followed by wet-mounting of a glass coverslip using 50% glycerol in 1× PBS. This helps to greatly reduce friction during coverslip removal, ultimately preserving the tissue structure between cycles. Images were acquired using the same microscope and objective as the H&E images. Slides were soaked in 42°C PBS to facilitate coverslip removal, and then fluorophores were inactivated by incubating slides in a solution of 4.5% H_2_O_2_ and 24 mmol/L NaOH in PBS and placing them under an LED light source for 1 hour. The list of all antibody panels used in the experiments is presented in Supplementary Table S1. Only antibodies that had passed a multi-step validation process and followed the expected staining pattern were included in downstream analysis.

### CyCIF image pre-processing and quality control

Stitching individual CyCIF images together into a high-dimensional representation for further segmentation and analyses was done using the open-source MCMICRO pipeline^52^ (RRID:SCR_022832), an open-source multiple-choice microscopy pipeline (version:38182748aa0ec021f684ce47248c57340d2f4cc7; full codes available on at https://github.com/labsyspharm/mcmicro. Specific parameters used were optimized after iterative inspection of results, specifically focused on performance of the segmentation module to ensure accurate identification of single cells (params.yml files available at https://github.com/labsyspharm/2025-Vallius-Shi-Novikov-melanoma-PCAII). After generating the segmentation masks, the mean fluorescence intensities of each marker for each cell were computed, resulting in a single-cell data table for each acquired whole-slide CyCIF image. The X/Y coordinates of annotated histologic regions on the whole-slide image were used to extract the single-cell data of cells that lie within the ROI range.

Multiple approaches were also taken to ensure the quality of the single-cell data. At the image level, the cross-cycle image registration and tissue integrity were reviewed; regions that were poorly registered or contained severely deformed tissues and artifacts were identified, and cells inside those regions were excluded. Antibodies that gave low confidence staining patterns by visual evaluation were also excluded from the analyses. The quality of the segmentation was assessed, and the segmentation parameters were iteratively modified to improve the accuracy of the segmentation masks.

### CyCIF single-cell phenotyping

We used a gating-based phenotyping approach to classify cells as described previously^10^. In short, an open-source visual gating tool (https://github.com/labsyspharm/gater) was used to derive gates for each marker. Similar to batch correction, the identified gates for each marker were then used to rescale the single-cell data between 0 and 1, such that the values above 0.5 identify cells that express the marker and vice versa. The scaled single-cell data was then used for cell-type calling. Tools within the SCIMAP^87^ python package (RRID:SCR_024751) were used to then assign phenotype labels to individual cells based on a hierarchical classification of staining patterns (Supplementary Fig. S1G, Fig. 2A,B, Supplementary Table S1,4). The assigned cell types were verified by overlaying the phenotypic labels onto the images.

### Correlation between protein markers and RNA levels

We performed correlation analysis between expression levels of melanoma gene expression, tumor state pathway scores, and CyCIF quantification of MRs across samples. The pairwise Spearman correlation coefficients were calculated based on the log-transformed RNA level, Protein expression level, and pathway scores, as well as CyCIF quantification of each GeoMX ROIs. pairwise correlation was calculated between those categories. Positive correlation was represented by red circles, with stronger correlations shown by darker and large circles. In contrast, negative correlations were represented by blue circles, with stronger negative correlations shown by darker and larger circles. Non-significant or weak correlations are smaller circles or absent circles near zero.

### Spatial entropy and identification of invasion bands

The local neighborhood Shannon entropy was calculated for each tumor cell according to its nearest neighbors defined via a Delaunay triangulation for the whole-slide images. In order to extend the notion of classical Shannon entropy to spatial entropy, spatial information was incorporated by forming a network graph G = (V, E) using the single cells per tissue specimen. The nodes V of the graph we defined according to the spatial location of single cells and edges E were specified via a Delaunay triangulation. Single-cell neighbors were defined by nearest neighbors in the resulting graph structure and edges were weighted by the Euclidean distance between single cells in tissue space. The graph G was also instantiated as a node attribute graph where each node (single-cell) was assigned the corresponding single cell phenotype (see Methods, CyCIF single-cell phenotyping). Numerous extensions and variations of entropy measures have been proposed in the literature^88,89^. The spatial entropy metric used here extends the intuition of Wang and Zhao^90^ in classifying landscapes in ecological studies whereby the Shannon entropy is augmented by a prefactor that is directly proportional to the intermixing strength and inversely proportional to the distance between nearest neighboring single cells of different phenotypes. The intermixing strength for each phenotype is defined as the cumulative total of the number of nearest neighbor single cells with differing phenotypes across all local neighborhoods. Given the scale differences between microscopy and ecological data, a scaling factor α is used here for visualization purposes. The form of spatial entropy employed in this work (is to the authors’ knowledge applied for the first time in the single-cell microscopy domain) was chosen for its objective and intuitive nature, as well as prior success in discerning spatial structure between varying ecological landscapes. The mathematical form of the spatial entropy H implemented in the single-cell analysis in this manuscript is detailed below:

$$H=\sum_{l=1}^{L}\frac{\alpha \frac{1}{n_{l}}\sum_{l=1}^{n_{l}}M_{i,l}}{\frac{1}{n_{l}}\sum_{i=1}^{n_{l}}D_{i,l}}p_{l}\;\log_{2}\;p_{l},$$

where the intermixing strength $M_{i,l}$ for cell i with attribute l is defined as,

$$M_{i,l}=\sum_{j=1}^{k_{i}}I\left(c_{j}\neq l\right)$$

and the distance $D_{i,l}$ for cell i with attribute l is defined as,

$$D_{i,l}=\sum_{j=1}^{k_{i}}d_{ij}I\left(c_{j}\neq l\right).$$

The variables and notation is defined as follows, L is the number of attributes, e.g., single-cell tumor phenotypes, $n_{l}$ is the number of single cells with attribute l in the tissue, $k_{i}$ is the number of nearest neighbors of cell i, $c_{j}$ is the attribute of cell j, $d_{ij}$ is the distance between cell i and cell j, I is the indicator function which equals 1 if the condition is satisfied and 0 otherwise, α is a scaling factor described above, and $p_{l}$ is the probability of attribute l in the tissue.

An open-source software package SpatialCells^91^ was used to identify subregions with a thickness of 0.2mm based on distance from the epidermis (invasion bands, Supplementary Fig. S7A). An increment of 0.2mm was used to separate each subregion. Local progression stages were further split into disjoint spatial subsections (in order not to bias the spatial analysis) and the spatial entropy H was calculated in each subregion.

### Recurrent cellular neighborhood (RCN) analysis

CyCIF neighborhood analyses were performed using the functions *spatial_count* and *spatial_cluster* within the SCIMAP^87^ python package. For each single cell in the CyCIF data, we determined the local neighborhood composition by querying a radius of 50 pixels (15 microns) from the cell centroid as measured by the Euclidean distance between X/Y coordinates. The cell types of these cellular neighbors and their frequencies were identified to generate a neighborhood matrix containing the neighbor phenotype information for every single cell in the data (*spatial_count*). The neighborhood matrix was then clustered using k-means clustering with *k* = 25 (*spatial_cluster*). The optimal number of *K*-means clustering was determined by looking for the elbow point in the computed cluster heterogeneity during convergence (Supplementary Fig. S12D). The clusters were then grouped into RCNGs (n=11) based on similarity of the microenvironmental composition. The RCNG assignments were visually confirmed by overlaying the RNCGs on multiple CyCIF images.

### Microregion transcriptomics (GeoMx^Ⓡ^) processing, data collection and annotation

For GeoMx digital spatial profiling (DSP), we used NanoString GeoMx human whole transcriptome atlas (WTA) RNA probes to profile the histopathological annotated regions mentioned above using previously described methods^49^. Briefly, freshly cut (< 2 weeks) 5 μm thick FFPE sections were baked at 60°C for 3 hours, dewaxed, and hybridized with the WTA probes overnight. On the following day, the slides were incubated with fluorescence-conjugated antibodies targeting melanocytes and tumor cells (SOX10/MLANA), epithelial cells (pan-CK), and immune cells (CD45) before imaging and transcript collection on the DSP (Supplementary Fig. S1D, Supplementary Table S1). These fluorescent signals were used for ROI selection for probe collection of 1,266 ROIs representing morphologically distinct sites (N, P, MIS, RGP, VGP; Fig. 1C). The collected probes were pooled for library preparation, and sent out for Next Generation Sequencing (Biopolymers Facility at Harvard Medical School). In addition to the annotations for local progression axis, the selected GeoMX microregions were annotated for location and immune responses. The annotations for location included epidermis (melanocyte and tumor regions in normal/precursor/MIS and the superficial intra-epidermal component above the RGP and VGP regions), tumor center, stroma, margin (invasive tumor front), and periphery (regions at the periphery of VGP either separate of the main tumor mass, or presenting with discohesive tumor cells at the periphery of VGP without a solid invasive front), tumor center, other). Immune responses for the microregions were annotated by visually assessing the CD45 staining and the cell morphology and were classified as brisk, non-brisk, or absent for RGP and VGP regions, and as immune response or no immune for N, P, and MIS regions.

### GeoMX trajectory analysis

Principal components analysis (PCA) was performed on the tumor (tumor/melanocytic annotated) MRs from all datasets (Sets A1, A2, B1, B2, e.g., Fig. 3A–I) without batch correction. Tumor MRs were projected onto the first two principal components (PC), withPC1 and PC2 explaining 28% and 14% of the variance, respectively. The inferred developmental trajectory, depicted as a black line, was computed using the *Slingshot* package (https://github.com/kstreet13/slingshot; RRID:SCR_017012) based on PC1 and PC2 embeddings. In addition, a separate trajectory analysis was performed on Set1 tumor MRs using PCA following batch correction with the *limma* package (e.g., Fig. 4A; RRID:SCR_010943).

### Differential gene expression and pathway analysis

Differential gene expression analysis was performed using the *DESeq2* package^92^ (RRID:SCR_015687). The top 60 differentially expressed genes (DEGs) were used for pathway enrichment analysis using the *fgsea* package (https://github.com/alserglab/fgsea) (e.g., in Fig. 6H). Additionally, normalized and log_10_-transformed expression matrices extracted from *DESeq2* were used to compute single-sample Gene Set Enrichment Analysis (ssGSEA) scores using the *GSVA* R package (https://www.bioconductor.org/packages/release/bioc/vignettes/GSVA/inst/doc/GSVA.html; RRID:SCR_021058; e.g., Fig. 5G).

### Self-organizing map (SOM) and the correlation spanning tree

Gene expression data from GeoMX Set A1 and Set A2 were log10-transformed, quantile normalized, and centralized on a gene-wise basis by subtracting the mean log expression of each gene (averaged across all samples) from its observed value. The processed data were then clustered using a self-organizing maps (SOMs) machine learning method^93^, resulting in 900 co-expressed metagenes (SOM was conducted within Set A1 and Set A2 separately due to batch effect issue). The so-called SOM-portrayal method was used to visualize the general gene expression levels of metagenes using a two-dimensional quadratic 30 × 30 pixel map and a maroon-to-blue color code for high-to-low metagene expression values^93,94^. Local progression stage -specific mean portraits were generated by averaging the metagene landscapes of all cases belonging to one category and difference portraits between them were calculated as the difference between the metagene values in eachgrid of these maps. Clusters of co-expressed metagenes were identified by selected so-called “cluster areas” in the SOM portraits using overexpression criteria as described previously^93^. The metagene Clusters (Fig. 5B and Supplementary Fig. S6C,F) were determined in unsupervised fashion following the SOM method previously described^93^.

A pairwise correlation analysis (Pearson’s correlation coefficient) was calculated using the metagene expression profiles among all tumor ROIs. The correlation matrix was then used to construct a correlation spanning tree, where each node represents an ROI and the edges represent the correlation strength between the ROIs. Based on the location of the nodes in the correlation spanning tree, each ROI was assigned a Branch ID (Branch 1, 2, or 3).

### Statistical Tests

All statistical tests to infer *P* value for significant differences in mean were performed using the MannWhitney U rank test with either *mannwhitneyu* function in the *scipy* Python package (RRID:SCR_008058) or *stats* and *ggpubr* R packages. The difference in the spatial entropy score variance within Breslow depth categories was tested with the Levene’s test using *car* R package.

## Supplementary Material

Supplement 1

Supplement 2

Supplement 3

Supplement 4

Supplement 5

Supplement 6

Supplement 7

## Data Availability

GeoMx gene expression data will be available via the Gene Expression Omnibus (GEO; RRID:SCR_005012). All CyCIF images with associated histopathological and GeoMX MR annotations will be available to explore online via Minerva software^84^, which supports zoom, pan, and selection actions without requiring the installation of software or downloading the images, at the Harvard Tissue Atlas (https://www.tissue-atlas.org/). Supplementary Video S1 gives an overview of how to navigate between channel groups and visualize annotations using Minerva. Selected CyCIF channels for specimen MEL14 can be explored via Minerva at https://www.cycif.org/data/vallius-2025/early_melanoma_tumor_intrinsic_e41/MEL14-A3/index.html. Full-resolution CyCIF images, single cell segmentation masks, and cell count tables will be available via the NCI Human Tumor Atlas Network data portal (https://data.humantumoratlas.org/); see Supplementary Table S1 for a mapping of sample numbers to HTAN IDs. Note that individual image files are ~100 GB in size. Code used for multimodal spatial analysis are available on GitHub (https://github.com/labsyspharm/2025-Vallius-Shi-Novikov-melanoma-PCAII ).

## References

[1] Arnold M.; Singh D.; Laversanne M.; Vignat J.; Vaccarella S.; Meheus F.; Cust A. E.; de Vries E.; Whiteman D. C.; Bray F. Global Burden of Cutaneous Melanoma in 2020 and Projections to 2040. JAMA Dermatol. 2022, 158 (5), 495–503. 10.1001/jamadermatol.2022.0160. 35353115 PMC8968696

[2] Domingues B.; Lopes J. M.; Soares P.; Pópulo H. Melanoma Treatment in Review. ImmunoTargets Ther. 2018, 7, 35–49. 10.2147/ITT.S134842. 29922629 PMC5995433

[3] Watts C. G.; McLoughlin K.; Goumas C.; van Kemenade C. H.; Aitken J. F.; Soyer H. P.; Fernandez Peñas P.; Guitera P.; Scolyer R. A.; Morton R. L.; Menzies S. W.; Caruana M.; Kang Y. J.; Mann G. J.; Chakera A. H.; Madronio C. M.; Armstrong B. K.; Thompson J. F.; Cust A. E. Association Between Melanoma Detected During Routine Skin Checks and Mortality. JAMA Dermatol. 2021, 157 (12), 1–12. 10.1001/jamadermatol.2021.3884. PMC8567188 34730781

[4] Keung E. Z.; Gershenwald J. E. The Eighth Edition American Joint Committee on Cancer (AJCC) Melanoma Staging System: Implications for Melanoma Treatment and Care. Expert Rev. Anticancer Ther. 2018, 18 (8), 775–784. 10.1080/14737140.2018.1489246. 29923435 PMC7652033

[5] Wan G.; Nguyen N.; Liu F.; DeSimone M. S.; Leung B. W.; Rajeh A.; Collier M. R.; Choi M. S.; Amadife M.; Tang K.; Zhang S.; Phillipps J. S.; Jairath R.; Alexander N. A.; Hua Y.; Jiao M.; Chen W.; Ho D.; Duey S.; Németh I. B.; Marko-Varga G.; Valdés J. G.; Liu D.; Boland G. M.; Gusev A.; Sorger P. K.; Yu K.-H.; Semenov Y. R. Prediction of Early-Stage Melanoma Recurrence Using Clinical and Histopathologic Features. NPJ Precis. Oncol. 2022, 6 (1), 79. 10.1038/s41698-022-00321-4. 36316482 PMC9622809

[6] Method of the Year 2024: Spatial Proteomics. Nat. Methods 2024, 21 (12), 2195–2196. 10.1038/s41592-024-02565-3. 39643689

[7] Tirosh I.; Izar B.; Prakadan S. M.; Wadsworth M. H.; Treacy D.; Trombetta J. J.; Rotem A.; Rodman C.; Lian C.; Murphy G.; Fallahi-Sichani M.; Dutton-Regester K.; Lin J.-R.; Cohen O.; Shah P.; Lu D.; Genshaft A. S.; Hughes T. K.; Ziegler C. G. K.; Kazer S. W.; Gaillard A.; Kolb K. E.; Villani A.-C.; Johannessen C. M.; Andreev A. Y.; Van Allen E. M.; Bertagnolli M.; Sorger P. K.; Sullivan R. J.; Flaherty K. T.; Frederick D. T.; Jané-Valbuena J.; Yoon C. H.; Rozenblatt-Rosen O.; Shalek A. K.; Regev A.; Garraway L. A. Dissecting the Multicellular Ecosystem of Metastatic Melanoma by Single-Cell RNA-Seq. Science 2016, 352 (6282), 189–196. 10.1126/science.aad0501. 27124452 PMC4944528

[8] Biermann J.; Melms J. C.; Amin A. D.; Wang Y.; Caprio L. A.; Karz A.; Tagore S.; Barrera I.; Ibarra-Arellano M. A.; Andreatta M.; Fullerton B. T.; Gretarsson K. H.; Sahu V.; Mangipudy V. S.; Nguyen T. T. T.; Nair A.; Rogava M.; Ho P.; Koch P. D.; Banu M.; Humala N.; Mahajan A.; Walsh Z. H.; Shah S. B.; Vaccaro D. H.; Caldwell B.; Mu M.; Wünnemann F.; Chazotte M.; Berhe S.; Luoma A. M.; Driver J.; Ingham M.; Khan S. A.; Rapisuwon S.; Slingluff C. L.; Eigentler T.; Röcken M.; Carvajal R.; Atkins M. B.; Davies M. A.; Agustinus A.; Bakhoum S. F.; Azizi E.; Siegelin M.; Lu C.; Carmona S. J.; Hibshoosh H.; Ribas A.; Canoll P.; Bruce J. N.; Bi W. L.; Agrawal P.; Schapiro D.; Hernando E.; Macosko E. Z.; Chen F.; Schwartz G. K.; Izar B. Dissecting the Treatment-Naive Ecosystem of Human Melanoma Brain Metastasis. Cell 2022, 185 (14), 2591–2608.e30. 10.1016/j.cell.2022.06.007. 35803246 PMC9677434

[9] Jerby-Arnon L.; Shah P.; Cuoco M. S.; Rodman C.; Su M.-J.; Melms J. C.; Leeson R.; Kanodia A.; Mei S.; Lin J.-R.; Wang S.; Rabasha B.; Liu D.; Zhang G.; Margolais C.; Ashenberg O.; Ott P. A.; Buchbinder E. I.; Haq R.; Hodi F. S.; Boland G. M.; Sullivan R. J.; Frederick D. T.; Miao B.; Moll T.; Flaherty K. T.; Herlyn M.; Jenkins R. W.; Thummalapalli R.; Kowalczyk M. S.; Cañadas I.; Schilling B.; Cartwright A. N. R.; Luoma A. M.; Malu S.; Hwu P.; Bernatchez C.; Forget M.-A.; Barbie D. A.; Shalek A. K.; Tirosh I.; Sorger P. K.; Wucherpfennig K.; Van Allen E. M.; Schadendorf D.; Johnson B. E.; Rotem A.; Rozenblatt-Rosen O.; Garraway L. A.; Yoon C. H.; Izar B.; Regev A. A Cancer Cell Program Promotes T Cell Exclusion and Resistance to Checkpoint Blockade. Cell 2018, 175 (4), 984–997.e24. 10.1016/j.cell.2018.09.006. 30388455 PMC6410377

[10] Nirmal A. J.; Maliga Z.; Vallius T.; Quattrochi B.; Chen A. A.; Jacobson C. A.; Pelletier R. J.; Yapp C.; Arias-Camison R.; Chen Y.-A.; Lian C. G.; Murphy G. F.; Santagata S.; Sorger P. K. The Spatial Landscape of Progression and Immunoediting in Primary Melanoma at Single-Cell Resolution. Cancer Discov. 2022, 12 (6), 1518–1541. 10.1158/2159-8290.CD-21-1357. 35404441 PMC9167783

[11] Shain A. H.; Yeh I.; Kovalyshyn I.; Sriharan A.; Talevich E.; Gagnon A.; Dummer R.; North J.; Pincus L.; Ruben B.; Rickaby W.; D’Arrigo C.; Robson A.; Bastian B. C. The Genetic Evolution of Melanoma from Precursor Lesions. N. Engl. J. Med. 2015, 373 (20), 1926–1936. 10.1056/NEJMoa1502583. 26559571

[12] Tang J.; Fewings E.; Chang D.; Zeng H.; Liu S.; Jorapur A.; Belote R. L.; McNeal A. S.; Tan T. M.; Yeh I.; Arron S. T.; Judson-Torres R. L.; Bastian B. C.; Shain A. H. The Genomic Landscapes of Individual Melanocytes from Human Skin. Nature 2020, 586 (7830), 600–605. 10.1038/s41586-020-2785-8. 33029006 PMC7581540

[13] Curtin J. A.; Fridlyand J.; Kageshita T.; Patel H. N.; Busam K. J.; Kutzner H.; Cho K.-H.; Aiba S.; Bröcker E.-B.; LeBoit P. E.; Pinkel D.; Bastian B. C. Distinct Sets of Genetic Alterations in Melanoma. N. Engl. J. Med. 2005, 353 (20), 2135–2147. 10.1056/NEJMoa050092. 16291983

[14] Galassi C.; Chan T. A.; Vitale I.; Galluzzi L. The Hallmarks of Cancer Immune Evasion. Cancer Cell 2024, 42 (11), 1825–1863. 10.1016/j.ccell.2024.09.010. 39393356

[15] Rambow F.; Marine J.-C.; Goding C. R. Melanoma Plasticity and Phenotypic Diversity: Therapeutic Barriers and Opportunities. Genes Dev. 2019, 33 (19–20), 1295–1318. 10.1101/gad.329771.119. 31575676 PMC6771388

[16] Martincorena I.; Roshan A.; Gerstung M.; Ellis P.; Van Loo P.; McLaren S.; Wedge D. C.; Fullam A.; Alexandrov L. B.; Tubio J. M.; Stebbings L.; Menzies A.; Widaa S.; Stratton M. R.; Jones P. H.; Campbell P. J. Tumor Evolution. High Burden and Pervasive Positive Selection of Somatic Mutations in Normal Human Skin. Science 2015, 348 (6237), 880–886. 10.1126/science.aaa6806. 25999502 PMC4471149

[17] Brash D. E. UV Signature Mutations. Photochem. Photobiol. 2015, 91 (1), 15–26. 10.1111/php.12377. 25354245 PMC4294947

[18] Long G. V.; Hauschild A.; Santinami M.; Kirkwood J. M.; Atkinson V.; Mandala M.; Merelli B.; Sileni V. C.; Nyakas M.; Haydon A.; Dutriaux C.; Robert C.; Mortier L.; Schachter J.; Schadendorf D.; Lesimple T.; Plummer R.; Larkin J.; Tan M.; Adnaik S. B.; Burgess P.; Jandoo T.; Dummer R. Final Results for Adjuvant Dabrafenib plus Trametinib in Stage III Melanoma. N. Engl. J. Med. 2024, 391 (18), 1709–1720. 10.1056/NEJMoa2404139. 38899716

[19] Liu D.; Lin J.-R.; Robitschek E. J.; Kasumova G. G.; Heyde A.; Shi A.; Kraya A.; Zhang G.; Moll T.; Frederick D. T.; Chen Y.-A.; Wang S.; Schapiro D.; Ho L.-L.; Bi K.; Sahu A.; Mei S.; Miao B.; Sharova T.; Alvarez-Breckenridge C.; Stocking J. H.; Kim T.; Fadden R.; Lawrence D.; Hoang M. P.; Cahill D. P.; Malehmir M.; Nowak M. A.; Brastianos P. K.; Lian C. G.; Ruppin E.; Izar B.; Herlyn M.; Van Allen E. M.; Nathanson K.; Flaherty K. T.; Sullivan R. J.; Kellis M.; Sorger P. K.; Boland G. M. Evolution of Delayed Resistance to Immunotherapy in a Melanoma Responder. Nat. Med. 2021, 27 (6), 985–992. 10.1038/s41591-021-01331-8. 33941922 PMC8474080

[20] Boshuizen J.; Vredevoogd D. W.; Krijgsman O.; Ligtenberg M. A.; Blankenstein S.; De Bruijn B.; Frederick D. T.; Kenski J. C. N.; Parren M.; Brüggemann M.; Madu M. F.; Rozeman E. A.; Song J.-Y.; Horlings H. M.; Blank C. U.; Van Akkooi A. C. J.; Flaherty K. T.; Boland G. M.; Peeper D. S. Reversal of Pre-Existing NGFR-Driven Tumor and Immune Therapy Resistance. Nat. Commun. 2020, 11 (1), 3946. 10.1038/s41467-020-17739-8. 32770055 PMC7414147

[21] Widmer D. S.; Cheng P. F.; Eichhoff O. M.; Belloni B. C.; Zipser M. C.; Schlegel N. C.; Javelaud D.; Mauviel A.; Dummer R.; Hoek K. S. Systematic Classification of Melanoma Cells by Phenotype-specific Gene Expression Mapping. Pigment Cell Melanoma Res. 2012, 25 (3), 343–353. 10.1111/j.1755-148X.2012.00986.x. 22336146

[22] Tsoi J.; Robert L.; Paraiso K.; Galvan C.; Sheu K. M.; Lay J.; Wong D. J. L.; Atefi M.; Shirazi R.; Wang X.; Braas D.; Grasso C. S.; Palaskas N.; Ribas A.; Graeber T. G. Multi-Stage Differentiation Defines Melanoma Subtypes with Differential Vulnerability to Drug-Induced Iron-Dependent Oxidative Stress. Cancer Cell 2018, 33 (5), 890–904.e5. 10.1016/j.ccell.2018.03.017. 29657129 PMC5953834

[23] Hoek K. S.; Schlegel N. C.; Brafford P.; Sucker A.; Ugurel S.; Kumar R.; Weber B. L.; Nathanson K. L.; Phillips D. J.; Herlyn M.; Schadendorf D.; Dummer R. Metastatic Potential of Melanomas Defined by Specific Gene Expression Profiles with No BRAF Signature. Pigment Cell Res. 2006, 19 (4), 290–302. 10.1111/j.1600-0749.2006.00322.x. 16827748

[24] Verfaillie A.; Imrichova H.; Atak Z. K.; Dewaele M.; Rambow F.; Hulselmans G.; Christiaens V.; Svetlichnyy D.; Luciani F.; Van Den Mooter L.; Claerhout S.; Fiers M.; Journe F.; Ghanem G.-E.; Herrmann C.; Halder G.; Marine J.-C.; Aerts S. Decoding the Regulatory Landscape of Melanoma Reveals TEADS as Regulators of the Invasive Cell State. Nat. Commun. 2015, 6 (1), 6683. 10.1038/ncomms7683. 25865119 PMC4403341

[25] Rambow F.; Rogiers A.; Marin-Bejar O.; Aibar S.; Femel J.; Dewaele M.; Karras P.; Brown D.; Chang Y. H.; Debiec-Rychter M.; Adriaens C.; Radaelli E.; Wolter P.; Bechter O.; Dummer R.; Levesque M.; Piris A.; Frederick D. T.; Boland G.; Flaherty K. T.; Van Den Oord J.; Voet T.; Aerts S.; Lund A. W.; Marine J.-C. Toward Minimal Residual Disease-Directed Therapy in Melanoma. Cell 2018, 174 (4), 843–855.e19. 10.1016/j.cell.2018.06.025. 30017245

[26] Hoek K. S.; Eichhoff O. M.; Schlegel N. C.; Döbbeling U.; Kobert N.; Schaerer L.; Hemmi S.; Dummer R. *In Vivo* Switching of Human Melanoma Cells between Proliferative and Invasive States. Cancer Res. 2008, 68 (3), 650–656. 10.1158/0008-5472.CAN-07-2491. 18245463

[27] Wouters J.; Kalender-Atak Z.; Minnoye L.; Spanier K. I.; De Waegeneer M.; Bravo González-Blas C.; Mauduit D.; Davie K.; Hulselmans G.; Najem A.; Dewaele M.; Pedri D.; Rambow F.; Makhzami S.; Christiaens V.; Ceyssens F.; Ghanem G.; Marine J.-C.; Poovathingal S.; Aerts S. Robust Gene Expression Programs Underlie Recurrent Cell States and Phenotype Switching in Melanoma. Nat. Cell Biol. 2020, 22 (8), 986–998. 10.1038/s41556-020-0547-3. 32753671

[28] Andrews M. C.; Oba J.; Wu C.-J.; Zhu H.; Karpinets T.; Creasy C. A.; Forget M.-A.; Yu X.; Song X.; Mao X.; Robertson A. G.; Romano G.; Li P.; Burton E. M.; Lu Y.; Sloane R. S.; Wani K. M.; Rai K.; Lazar A. J.; Haydu L. E.; Bustos M. A.; Shen J.; Chen Y.; Morgan M. B.; Wargo J. A.; Kwong L. N.; Haymaker C. L.; Grimm E. A.; Hwu P.; Hoon D. S. B.; Zhang J.; Gershenwald J. E.; Davies M. A.; Futreal P. A.; Bernatchez C.; Woodman S. E. Multi-Modal Molecular Programs Regulate Melanoma Cell State. Nat. Commun. 2022, 13 (1), 4000. 10.1038/s41467-022-31510-1. 35810190 PMC9271073

[29] Landsberg J.; Kohlmeyer J.; Renn M.; Bald T.; Rogava M.; Cron M.; Fatho M.; Lennerz V.; Wölfel T.; Hölzel M.; Tüting T. Melanomas Resist T-Cell Therapy through Inflammation-Induced Reversible Dedifferentiation. Nature 2012, 490 (7420), 412–416. 10.1038/nature11538. 23051752

[30] Kim Y. J.; Sheu K. M.; Tsoi J.; Abril-Rodriguez G.; Medina E.; Grasso C. S.; Torrejon D. Y.; Champhekar A. S.; Litchfield K.; Swanton C.; Speiser D. E.; Scumpia P. O.; Hoffmann A.; Graeber T. G.; Puig-Saus C.; Ribas A. Melanoma Dedifferentiation Induced by IFN-γ Epigenetic Remodeling in Response to Anti-PD-1 Therapy. J. Clin. Invest. 2021, 131 (12), e145859, 145859. 10.1172/JCI145859. 33914706 PMC8203459

[31] Widmer D. S.; Hoek K. S.; Cheng P. F.; Eichhoff O. M.; Biedermann T.; Raaijmakers M. I. G.; Hemmi S.; Dummer R.; Levesque M. P. Hypoxia Contributes to Melanoma Heterogeneity by Triggering HIF1α-Dependent Phenotype Switching. J. Invest. Dermatol. 2013, 133 (10), 2436–2443. 10.1038/jid.2013.115. 23474946

[32] Mehta A.; Kim Y. J.; Robert L.; Tsoi J.; Comin-Anduix B.; Berent-Maoz B.; Cochran A. J.; Economou J. S.; Tumeh P. C.; Puig-Saus C.; Ribas A. Immunotherapy Resistance by Inflammation-Induced Dedifferentiation. Cancer Discov. 2018, 8 (8), 935–943. 10.1158/2159-8290.CD-17-1178. 29899062 PMC6076867

[33] Hoek K. S.; Eichhoff O. M.; Schlegel N. C.; Döbbeling U.; Kobert N.; Schaerer L.; Hemmi S.; Dummer R. *In Vivo* Switching of Human Melanoma Cells between Proliferative and Invasive States. Cancer Res. 2008, 68 (3), 650–656. 10.1158/0008-5472.CAN-07-2491. 18245463

[34] Caramel J.; Papadogeorgakis E.; Hill L.; Browne G. J.; Richard G.; Wierinckx A.; Saldanha G.; Osborne J.; Hutchinson P.; Tse G.; Lachuer J.; Puisieux A.; Pringle J. H.; Ansieau S.; Tulchinsky E. A Switch in the Expression of Embryonic EMT-Inducers Drives the Development of Malignant Melanoma. Cancer Cell 2013, 24 (4), 466–480. 10.1016/j.ccr.2013.08.018. 24075834

[35] Hoashi T.; Watabe H.; Muller J.; Yamaguchi Y.; Vieira W. D.; Hearing V. J. MART-1 Is Required for the Function of the Melanosomal Matrix Protein PMEL17/GP100 and the Maturation of Melanosomes. J. Biol. Chem. 2005, 280 (14), 14006–14016. 10.1074/jbc.M413692200. 15695812

[36] Kapur R. P.; Bigler S. A.; Skelly M.; Gown A. M. Anti-Melanoma Monoclonal Antibody HMB45 Identifies an Oncofetal Glycoconjugate Associated with Immature Melanosomes. J. Histochem. Cytochem. 1992, 40 (2), 207–212. 10.1177/40.2.1552165. 1552165

[37] Ohsie S. J.; Sarantopoulos G. P.; Cochran A. J.; Binder S. W. Immunohistochemical Characteristics of Melanoma. J. Cutan. Pathol. 2008, 35 (5), 433–444. 10.1111/j.1600-0560.2007.00891.x. 18399807

[38] Kim R. H.; Meehan S. A. Immunostain Use in the Diagnosis of Melanomas Referred to a Tertiary Medical Center: A 15-year Retrospective Review (2001–2015). J. Cutan. Pathol. 2017, 44 (3), 221–227. 10.1111/cup.12867. 27873341

[39] Berset M.; Cerottini J. P.; Guggisberg D.; Romero P.; Burri F.; Rimoldi D.; Panizzon R. G. Expression of Melan-A/MART-1 Antigen as a Prognostic Factor in Primary Cutaneous Melanoma. Int. J. Cancer 2001, 95 (1), 73–77. 10.1002/1097-0215(20010120)95:1<73::aid-ijc1013>3.0.co;2-s. 11241315

[40] Cronin J. C.; Watkins-Chow D. E.; Incao A.; Hasskamp J. H.; Schönewolf N.; Aoude L. G.; Hayward N. K.; Bastian B. C.; Dummer R.; Loftus S. K.; Pavan W. J. SOX10 Ablation Arrests Cell Cycle, Induces Senescence, and Suppresses Melanomagenesis. Cancer Res. 2013, 73 (18), 5709–5718. 10.1158/0008-5472.CAN-12-4620. 23913827 PMC3803156

[41] Laga A. C.; Murphy G. F. Cellular Heterogeneity in Vertical Growth Phase Melanoma. Arch. Pathol. Lab. Med. 2010, 134 (12), 1750–1757. 10.5858/2009-0394-RAR.1. 21128771

[42] Gershenwald J. E.; Scolyer R. A.; Hess K. R.; Sondak V. K.; Long G. V.; Ross M. I.; Lazar A. J.; Faries M. B.; Kirkwood J. M.; McArthur G. A.; Haydu L. E.; Eggermont A. M. M.; Flaherty K. T.; Balch C. M.; Thompson J. F.; for members of the American Joint Committee on Cancer Melanoma Expert Panel and the International Melanoma Database and Discovery Platform. Melanoma Staging: Evidence-based Changes in the American Joint Committee on Cancer Eighth Edition Cancer Staging Manual. CA. Cancer J. Clin. 2017, 67 (6), 472–492. 10.3322/caac.21409. 29028110 PMC5978683

[43] Maibach F.; Sadozai H.; Seyed Jafari S. M.; Hunger R. E.; Schenk M. Tumor-Infiltrating Lymphocytes and Their Prognostic Value in Cutaneous Melanoma. Front. Immunol. 2020, 11. 10.3389/fimmu.2020.02105. PMC7511547 33013886

[44] Pozniak J.; Pedri D.; Landeloos E.; Van Herck Y.; Antoranz A.; Vanwynsberghe L.; Nowosad A.; Roda N.; Makhzami S.; Bervoets G.; Maciel L. F.; Pulido-Vicuña C. A.; Pollaris L.; Seurinck R.; Zhao F.; Flem-Karlsen K.; Damsky W.; Chen L.; Karagianni D.; Cinque S.; Kint S.; Vandereyken K.; Rombaut B.; Voet T.; Vernaillen F.; Annaert W.; Lambrechts D.; Boecxstaens V.; Saeys Y.; Van Den Oord J.; Bosisio F.; Karras P.; Shain A. H.; Bosenberg M.; Leucci E.; Paschen A.; Rambow F.; Bechter O.; Marine J.-C. A TCF4-Dependent Gene Regulatory Network Confers Resistance to Immunotherapy in Melanoma. Cell 2024, 187 (1), 166–183.e25. 10.1016/j.cell.2023.11.037. 38181739

[45] Yapp C.; Nirmal A. J.; Zhou F. Y.; Wong A. Y. H.; Tefft J.; Lu Y. D.; Shang Z.; Maliga Z.; Llopis P. M.; Murphy G. F.; Lian C.; Danuser G.; Santagata S.; Sorger P. K. Highly Multiplexed 3D Profiling of Cell States and Immune Niches in Human Tumours. bioRxiv April 11, 2025, p 2023.11.10.566670. 10.1101/2023.11.10.566670. PMC12510885 41023436

[46] Ennen M.; Keime C.; Gambi G.; Kieny A.; Coassolo S.; Thibault-Carpentier C.; Margerin-Schaller F.; Davidson G.; Vagne C.; Lipsker D.; Davidson I. *MITF* -High and *MITF* -Low Cells and a Novel Subpopulation Expressing Genes of Both Cell States Contribute to Intra- and Intertumoral Heterogeneity of Primary Melanoma. Clin. Cancer Res. 2017, 23 (22), 7097–7107. 10.1158/1078-0432.CCR-17-0010. 28855355

[47] Kunz M.; Löffler-Wirth H.; Dannemann M.; Willscher E.; Doose G.; Kelso J.; Kottek T.; Nickel B.; Hopp L.; Landsberg J.; Hoffmann S.; Tüting T.; Zigrino P.; Mauch C.; Utikal J.; Ziemer M.; Schulze H.-J.; Hölzel M.; Roesch A.; Kneitz S.; Meierjohann S.; Bosserhoff A.; Binder H.; Schartl M. RNA-Seq Analysis Identifies Different Transcriptomic Types and Developmental Trajectories of Primary Melanomas. Oncogene 2018, 37 (47), 6136–6151. 10.1038/s41388-018-0385-y. 29995873

[48] Lin J.-R.; Izar B.; Wang S.; Yapp C.; Mei S.; Shah P. M.; Santagata S.; Sorger P. K. Highly Multiplexed Immunofluorescence Imaging of Human Tissues and Tumors Using T-CyCIF and Conventional Optical Microscopes. eLife 2018, 7, e31657. 10.7554/eLife.31657. 29993362 PMC6075866

[49] Zollinger D. R.; Lingle S. E.; Sorg K.; Beechem J. M.; Merritt C. R. GeoMx™ RNA Assay: High Multiplex, Digital, Spatial Analysis of RNA in FFPE Tissue. Methods Mol. Biol. Clifton NJ 2020, 2148, 331–345. 10.1007/978-1-0716-0623-0_21. 32394392

[50] Lin J.-R.; Wang S.; Coy S.; Chen Y.-A.; Yapp C.; Tyler M.; Nariya M. K.; Heiser C. N.; Lau K. S.; Santagata S.; Sorger P. K. Multiplexed 3D Atlas of State Transitions and Immune Interaction in Colorectal Cancer. Cell 2023, 186 (2), 363–381.e19. 10.1016/j.cell.2022.12.028. 36669472 PMC10019067

[51] Baker G. J.; Novikov E.; Zhao Z.; Vallius T.; Davis J. A.; Lin J.-R.; Muhlich J. L.; Mittendorf E. A.; Santagata S.; Guerriero J. L.; Sorger P. K. Quality Control for Single-Cell Analysis of High-Plex Tissue Profiles Using CyLinter. Nat. Methods 2024, 21 (12), 2248–2259. 10.1038/s41592-024-02328-0. 39478175 PMC11621021

[52] Schapiro D.; Sokolov A.; Yapp C.; Chen Y.-A.; Muhlich J. L.; Hess J.; Creason A. L.; Nirmal A. J.; Baker G. J.; Nariya M. K.; Lin J.-R.; Maliga Z.; Jacobson C. A.; Hodgman M. W.; Ruokonen J.; Farhi S. L.; Abbondanza D.; McKinley E. T.; Persson D.; Betts C.; Sivagnanam S.; Regev A.; Goecks J.; Coffey R. J.; Coussens L. M.; Santagata S.; Sorger P. K. MCMICRO: A Scalable, Modular Image-Processing Pipeline for Multiplexed Tissue Imaging. Nat. Methods 2022, 19 (3), 311–315. 10.1038/s41592-021-01308-y. 34824477 PMC8916956

[53] Jönsson G.; Busch C.; Knappskog S.; Geisler J.; Miletic H.; Ringnér M.; Lillehaug J. R.; Borg Å.; Lønning P. E. Gene Expression Profiling–Based Identification of Molecular Subtypes in Stage IV Melanomas with Different Clinical Outcome. Clin. Cancer Res. 2010, 16 (13), 3356–3367. 10.1158/1078-0432.CCR-09-2509. 20460471

[54] Harbst K.; Staaf J.; Lauss M.; Karlsson A.; Måsbäck A.; Johansson I.; Bendahl P.-O.; Vallon-Christersson J.; Törngren T.; Ekedahl H.; Geisler J.; Höglund M.; Ringnér M.; Lundgren L.; Jirström K.; Olsson H.; Ingvar C.; Borg Å.; Tsao H.; Jönsson G. Molecular Profiling Reveals Low- and High-Grade Forms of Primary Melanoma. Clin. Cancer Res. 2012, 18 (15), 4026–4036. 10.1158/1078-0432.CCR-12-0343. 22675174 PMC3467105

[55] Thompson J. F.; Soong S.-J.; Balch C. M.; Gershenwald J. E.; Ding S.; Coit D. G.; Flaherty K. T.; Gimotty P. A.; Johnson T.; Johnson M. M.; Leong S. P.; Ross M. I.; Byrd D. R.; Cascinelli N.; Cochran A. J.; Eggermont A. M.; McMasters K. M.; Mihm M. C.; Morton D. L.; Sondak V. K. Prognostic Significance of Mitotic Rate in Localized Primary Cutaneous Melanoma: An Analysis of Patients in the Multi-Institutional American Joint Committee on Cancer Melanoma Staging Database. J. Clin. Oncol. 2011, 29 (16), 2199–2205. 10.1200/JCO.2010.31.5812. 21519009 PMC3107741

[56] Gimotty P. A.; Van Belle P.; Elder D. E.; Murry T.; Montone K. T.; Xu X.; Hotz S.; Raines S.; Ming M. E.; Wahl P.; Guerry D. Biologic and Prognostic Significance of Dermal Ki67 Expression, Mitoses, and Tumorigenicity in Thin Invasive Cutaneous Melanoma. J. Clin. Oncol. 2005, 23 (31), 8048–8056. 10.1200/JCO.2005.02.0735. 16258103

[57] Du J.; Miller A. J.; Widlund H. R.; Horstmann M. A.; Ramaswamy S.; Fisher D. E. MLANA/MART1 and SILV/PMEL17/GP100 Are Transcriptionally Regulated by MITF in Melanocytes and Melanoma. Am. J. Pathol. 2003, 163 (1), 333–343. 10.1016/S0002-9440(10)63657-7. 12819038 PMC1868174

[58] Fetsch P. A.; Marincola F. M.; Filie A.; Hijazi Y. M.; Kleiner D. E.; Abati A. Melanoma-Associated Antigen Recognized by T Cells (MART-1): The Advent of a Preferred Immunocytochemical Antibody for the Diagnosis of Metastatic Malignant Melanoma with Fine-Needle Aspiration. Cancer 1999, 87 (1), 37–42. 10096358

[59] Qiu W.; Chuong C.-M.; Lei M. Regulation of Melanocyte Stem Cells in the Pigmentation of Skin and Its Appendages: Biological Patterning and Therapeutic Potentials. Exp. Dermatol. 2019, 28 (4), 395–405. 10.1111/exd.13856. 30537004 PMC6488374

[60] Gopalan V.; Day C.-P.; Pérez-Guijarro E.; Chin S.; Ebersole J.; Smith C.; Simpson M.; Sassano A.; Constantino M. A.; Wu E.; Yang H. H.; Lee M. P.; Hannenhalli S.; Merlino G.; Marie K. L. Comprehensive Single-Cell Transcriptomic Analysis of Embryonic Melanoblasts Uncovers Lineage-Specific Mechanisms of Melanoma Metastasis and Therapy Resistance; preprint; Cancer Biology, 2022. 10.1101/2022.10.14.512297.

[61] Zhang Z.; Imani S.; Shasaltaneh M. D.; Hosseinifard H.; Zou L.; Fan Y.; Wen Q. The Role of Vascular Mimicry as a Biomarker in Malignant Melanoma: A Systematic Review and Meta-Analysis. BMC Cancer 2019, 19 (1), 1134. 10.1186/s12885-019-6350-5. 31752759 PMC6873453

[62] Mihm M. C.; Mulé J. J. Reflections on the Histopathology of Tumor-Infiltrating Lymphocytes in Melanoma and the Host Immune Response. Cancer Immunol. Res. 2015, 3 (8), 827–835. 10.1158/2326-6066.CIR-15-0143. 26242760 PMC4527084

[63] Cugno M.; Borghi A.; Garcovich S.; Marzano A. V. Coagulation and Skin Autoimmunity. Front. Immunol. 2019, 10. 10.3389/fimmu.2019.01407. PMC6596352 31281319

[64] Xu Y.; Olman V.; Xu D. Minimum Spanning Trees for Gene Expression Data Clustering. Genome Inform. Int. Conf. Genome Inform. 2001, 12, 24–33. 11791221

[65] Srivastava A.; Laidler P.; Davies R. P.; Horgan K.; Hughes L. E. The Prognostic Significance of Tumor Vascularity in Intermediate-Thickness (0.76–4.0 Mm Thick) Skin Melanoma. A Quantitative Histologic Study. Am. J. Pathol. 1988, 133 (2), 419–423. 3189515 PMC1880778

[66] Weinstein D.; Leininger J.; Hamby C.; Safai B. Diagnostic and Prognostic Biomarkers in Melanoma. J. Clin. Aesthetic Dermatol. 2014, 7 (6), 13–24. PMC4086529 25013535

[67] Bai X.; Fisher D. E.; Flaherty K. T. Cell-State Dynamics and Therapeutic Resistance in Melanoma from the Perspective of MITF and IFNγ Pathways. Nat. Rev. Clin. Oncol. 2019, 16 (9), 549–562. 10.1038/s41571-019-0204-6. 30967646 PMC7185899

[68] Hölzel M.; Tüting T. Inflammation-Induced Plasticity in Melanoma Therapy and Metastasis. Trends Immunol. 2016, 37 (6), 364–374. 10.1016/j.it.2016.03.009. 27151281

[69] Fu Q.; Chen N.; Ge C.; Li R.; Li Z.; Zeng B.; Li C.; Wang Y.; Xue Y.; Song X.; Li H.; Li G. Prognostic Value of Tumor-Infiltrating Lymphocytes in Melanoma: A Systematic Review and Meta-Analysis. Oncoimmunology 2019, 8 (7), 1593806. 10.1080/2162402X.2019.1593806. 31143514 PMC6527267

[70] Smith M. P.; Sanchez-Laorden B.; O’Brien K.; Brunton H.; Ferguson J.; Young H.; Dhomen N.; Flaherty K. T.; Frederick D. T.; Cooper Z. A.; Wargo J. A.; Marais R.; Wellbrock C. The Immune Microenvironment Confers Resistance to MAPK Pathway Inhibitors through Macrophage-Derived TNFα. Cancer Discov. 2014, 4 (10), 1214–1229. 10.1158/2159-8290.CD-13-1007. 25256614 PMC4184867

[71] Lugassy C.; Kleinman H. K.; Vermeulen P. B.; Barnhill R. L. Angiotropism, Pericytic Mimicry and Extravascular Migratory Metastasis: An Embryogenesis-Derived Program of Tumor Spread. Angiogenesis 2020, 23 (1), 27–41. 10.1007/s10456-019-09695-9. 31720876

[72] Chu X.; Tian W.; Ning J.; Xiao G.; Zhou Y.; Wang Z.; Zhai Z.; Tanzhu G.; Yang J.; Zhou R. Cancer Stem Cells: Advances in Knowledge and Implications for Cancer Therapy. Signal Transduct. Target. Ther. 2024, 9, 170. 10.1038/s41392-024-01851-y. 38965243 PMC11224386

[73] Braverman I. M. The Cutaneous Microcirculation. J. Investig. Dermatol. Symp. Proc. 2000, 5 (1), 3–9. 10.1046/j.1087-0024.2000.00010.x. 11147672

[74] Nociceptor neurons affect cancer immunosurveillance | Nature. https://www.nature.com/articles/s41586-022-05374-w (accessed 2024-06-30). 10.1038/s41586-022-05374-w PMC9646485 36323780

[75] Pozdnyakova O.; Grossman J.; Barbagallo B.; Lyle S. The Hair Follicle Barrier to Involvement by Malignant Melanoma. Cancer 2009, 115 (6), 1267–1275. 10.1002/cncr.24117. 19152437

[76] Grossman D.; Okwundu N.; Bartlett E. K.; Marchetti M. A.; Othus M.; Coit D. G.; Hartman R. I.; Leachman S. A.; Berry E. G.; Korde L.; Lee S. J.; Bar-Eli M.; Berwick M.; Bowles T.; Buchbinder E. I.; Burton E. M.; Chu E. Y.; Curiel-Lewandrowski C.; Curtis J. A.; Daud A.; Deacon D. C.; Ferris L. K.; Gershenwald J. E.; Grossmann K. F.; Hu-Lieskovan S.; Hyngstrom J.; Jeter J. M.; Judson-Torres R. L.; Kendra K. L.; Kim C. C.; Kirkwood J. M.; Lawson D. H.; Leming P. D.; Long G. V.; Marghoob A. A.; Mehnert J. M.; Ming M. E.; Nelson K. C.; Polsky D.; Scolyer R. A.; Smith E. A.; Sondak V. K.; Stark M. S.; Stein J. A.; Thompson J. A.; Thompson J. F.; Venna S. S.; Wei M. L.; Swetter S. M. Prognostic Gene Expression Profiling in Cutaneous Melanoma: Identifying the Knowledge Gaps and Assessing the Clinical Benefit. JAMA Dermatol. 2020, 156 (9), 1004. 10.1001/jamadermatol.2020.1729. 32725204 PMC8275355

[77] Manton R. N.; Roshan A. Systematic Review of Risk Prediction Tools for Primary Cutaneous Melanoma Outcomes and Validation of Sentinel Lymph Node Positivity Prediction in a UK Tertiary Cohort. BJC Rep. 2024, 2 (1), 86. 10.1038/s44276-024-00110-5. 39528626 PMC11554800

[78] Stassen R. C.; Mulder E. E. A. P.; Mooyaart A. L.; Francken A. B.; van der Hage J.; Aarts M. J. B.; van der Veldt A. A. M.; Verhoef C.; Grünhagen D. J. Clinical Evaluation of the Clinicopathologic and Gene Expression Profile (CP-GEP) in Patients with Melanoma Eligible for Sentinel Lymph Node Biopsy: A Multicenter Prospective Dutch Study. Eur. J. Surg. Oncol. J. Eur. Soc. Surg. Oncol. Br. Assoc. Surg. Oncol. 2023, 49 (12), 107249. 10.1016/j.ejso.2023.107249. 37907016

[79] Risk stratification using the Merlin Assay (CP-GEP) in an independent cohort of 930 patients with clinical stage I/II melanoma who did not undergo sentinel lymph node biopsy - PubMed. https://pubmed-ncbi-nlm-nih-gov.ezp-prod1.hul.harvard.edu/40274320/ (accessed 2025-06-07). 10.1016/j.ejca.2025.115372 40274320

[80] Cook R. W.; Middlebrook B.; Wilkinson J.; Covington K. R.; Oelschlager K.; Monzon F. A.; Stone J. F. Analytic Validity of DecisionDx-Melanoma, a Gene Expression Profile Test for Determining Metastatic Risk in Melanoma Patients. Diagn. Pathol. 2018, 13 (1), 13. 10.1186/s13000-018-0690-3. 29433548 PMC5809902

[81] Ziemer M.; Weidenthaler-Barth B.; Gussek P.; Pfeiffer M.; Kleemann J.; Bankov K.; Wild P. J.; Seibold S.; Sureshkumar P.; Nickel P.; Strobel A.; Werner M.; Grabbe S. Analytical Validation of an Immunohistochemical 7-Biomarker Prognostic Assay (Immunoprint®) for Early-Stage Cutaneous Melanoma in Archival Tissue of Patients with AJCC v8 T2–T3 Disease. Diagnostics 2023, 13 (19), 3096. 10.3390/diagnostics13193096. 37835839 PMC10572486

[82] Chan W. H.; Tsao H. Consensus, Controversy, and Conversations About Gene Expression Profiling in Melanoma. JAMA Dermatol. 2020, 156 (9), 949–951. 10.1001/jamadermatol.2020.1730. 32745178

[83] Sun J.; Karasaki K. M.; Farma J. M. The Use of Gene Expression Profiling and Biomarkers in Melanoma Diagnosis and Predicting Recurrence: Implications for Surveillance and Treatment. Cancers 2024, 16 (3), 583. 10.3390/cancers16030583. 38339333 PMC10854922

[84] Hoffer J.; Rashid R.; Muhlich J.; Chen Y.-A.; Russell D.; Ruokonen J.; Krueger R.; Pfister H.; Santagata S.; Sorger P. Minerva: A Light-Weight, Narrative Image Browser for Multiplexed Tissue Images. J. Open Source Softw. 2020, 5 (54), 2579. 10.21105/joss.02579. 33768192 PMC7989801

[85] Schapiro D.; Yapp C.; Sokolov A.; Reynolds S. M.; Chen Y.-A.; Sudar D.; Xie Y.; Muhlich J.; Arias-Camison R.; Arena S.; Taylor A. J.; Nikolov M.; Tyler M.; Lin J.-R.; Burlingame E. A.; Human Tumor Atlas Network; Chang Y. H.; Farhi S. L.; Thorsson V.; Venkatamohan N.; Drewes J. L.; Pe’er D.; Gutman D. A.; Herrmann M. D.; Gehlenborg N.; Bankhead P.; Roland J. T.; Herndon J. M.; Snyder M. P.; Angelo M.; Nolan G.; Swedlow J. R.; Schultz N.; Merrick D. T.; Mazzili S. A.; Cerami E.; Rodig S. J.; Santagata S.; Sorger P. K. MITI Minimum Information Guidelines for Highly Multiplexed Tissue Images. Nat. Methods 2022, 19 (3), 262–267. 10.1038/s41592-022-01415-4. 35277708 PMC9009186

[86] Lin J.-R.; Izar B.; Wang S.; Yapp C.; Mei S.; Shah P. M.; Santagata S.; Sorger P. K. Highly Multiplexed Immunofluorescence Imaging of Human Tissues and Tumors Using T-CyCIF and Conventional Optical Microscopes. eLife 2018, 7, e31657. 10.7554/eLife.31657. 29993362 PMC6075866

[87] Nirmal A. J.; Sorger P. K. SCIMAP: A Python Toolkit for Integrated SpatialAnalysis of Multiplexed Imaging Data. J. Open Source Softw. 2024, 9 (97), 6604. 10.21105/joss.06604. 38873023 PMC11173324

[88] Altieri L.; Cocchi D.; Roli G. Efficient Computation of Spatial Entropy Measures. Entropy 2023, 25 (12), 1634. 10.3390/e25121634. 38136514 PMC10742711

[89] Batty M.; Morphet R.; Masucci P.; Stanilov K. Entropy, Complexity, and Spatial Information. J. Geogr. Syst. 2014, 16 (4), 363–385. 10.1007/s10109-014-0202-2. 25309123 PMC4179993

[90] Wang C.; Zhao H. Spatial Heterogeneity Analysis: Introducing a New Form of Spatial Entropy. Entropy 2018, 20 (6), 398. 10.3390/e20060398. 33265488 PMC7512918

[91] Wan G.; Maliga Z.; Yan B.; Vallius T.; Shi Y.; Khattab S.; Chang C.; Nirmal A. J.; Yu K.-H.; Liu D.; Lian C. G.; DeSimone M. S.; Sorger P. K.; Semenov Y. R. SpatialCells: Automated Profiling of Tumor Microenvironments with Spatially Resolved Multiplexed Single-Cell Data. Brief. Bioinform. 2024, 25 (3), bbae189. 10.1093/bib/bbae189. 38701421 PMC11066940

[92] Love M. I.; Huber W.; Anders S. Moderated Estimation of Fold Change and Dispersion for RNA-Seq Data with DESeq2. Genome Biol. 2014, 15 (12), 550. 10.1186/s13059-014-0550-8. 25516281 PMC4302049

[93] Wirth H.; Löffler M.; Von Bergen M.; Binder H. Expression Cartography of Human Tissues Using Self Organizing Maps. BMC Bioinformatics 2011, 12 (1), 306. 10.1186/1471-21-05-12-306. 21794127 PMC3161046

[94] Wirth H.; Von Bergen M.; Binder H. Mining SOM Expression Portraits: Feature Selection and Integrating Concepts of Molecular Function. BioData Min. 2012, 5 (1), 18. 10.1186/1756-0381-5-18. 23043905 PMC3599960

